# Structure of the DP1–DP2 PolD complex bound with DNA and its implications for the evolutionary history of DNA and RNA polymerases

**DOI:** 10.1371/journal.pbio.3000122

**Published:** 2019-01-18

**Authors:** Pierre Raia, Marta Carroni, Etienne Henry, Gérard Pehau-Arnaudet, Sébastien Brûlé, Pierre Béguin, Ghislaine Henneke, Erik Lindahl, Marc Delarue, Ludovic Sauguet

**Affiliations:** 1 Unit of Structural Dynamics of Macromolecules, Pasteur Institute and CNRS UMR 3528, Paris, France; 2 Sorbonne Université, Ecole Doctorale Complexité du Vivant (ED515), Paris, France; 3 Science for Life Laboratory, Department of Biochemistry and Biophysics, Stockholm University, Sweden; 4 CNRS, IFREMER, Univ Brest, Laboratoire de Microbiologie des Environnements Extrêmes, Plouzané, France; 5 Utech UBI, Pasteur Institute and CNRS UMR 3528, Paris, France; 6 Molecular Biophysics Platform, Pasteur Institute, C2RT and CNRS UMR 3528, Paris, France; 7 Unit of Molecular Biology of Gene in Extremophiles, Pasteur Institute, Paris, France; 8 IFREMER, CNRS, Univ Brest, Laboratoire de Microbiologie des Environnements Extrêmes, Plouzané, France; Rutgers University-Robert Wood Johnson Medical School, UNITED STATES

## Abstract

PolD is an archaeal replicative DNA polymerase (DNAP) made of a proofreading exonuclease subunit (DP1) and a larger polymerase catalytic subunit (DP2). Recently, we reported the individual crystal structures of the DP1 and DP2 catalytic cores, thereby revealing that PolD is an atypical DNAP that has all functional properties of a replicative DNAP but with the catalytic core of an RNA polymerase (RNAP). We now report the DNA-bound cryo–electron microscopy (cryo-EM) structure of the heterodimeric DP1–DP2 PolD complex from *Pyrococcus abyssi*, revealing a unique DNA-binding site. Comparison of PolD and RNAPs extends their structural similarities and brings to light the minimal catalytic core shared by all cellular transcriptases. Finally, elucidating the structure of the PolD DP1–DP2 interface, which is conserved in all eukaryotic replicative DNAPs, clarifies their evolutionary relationships with PolD and sheds light on the domain acquisition and exchange mechanism that occurred during the evolution of the eukaryotic replisome.

## Abstract 2

A cryo-EM reconstruction of the archaeal PolD DNA polymerase assembly bound to DNA defines the minimal catalytic fold for two-barrel DNA and RNA polymerases and suggests a scenario for the evolution of the eukaryotic replicative apparatus.

## Author summary

PolD is an unusual archaeal replicative DNA polymerase (DNAP) in that it has all the functional properties of a replicative DNAP but with a catalytic core reminiscent of an RNA polymerase (RNAP). We now describe the cryo–electron microscopy (cryo-EM) structure of a DNA-bound heterodimeric DP1–DP2 PolD complex from the archaeon *Pyrococcus abyssi*, revealing a unique DNA-binding site. Comparison of PolD with RNAPs extends their structural similarities and highlights the minimal catalytic core shared by all cellular transcriptases. Elucidating the structure of the interface between PolD’s DP1 and DP2 subunits, which is conserved in all eukaryotic replicative DNA polymerases, clarifies their evolutionary relationships with PolD and sheds light on the process of domain acquisition and exchange that occurred during the evolution of the eukaryotic replisome.

## Introduction

All forms of life have evolved multiple DNA polymerases (DNAPs) in order to maintain their genomes [1]. Highly processive and accurate replicative DNAPs are responsible for duplicating the genome, and a variety of specialized DNAPs are involved in DNA repair and in resolving the Okazaki fragments. Over the years, all DNAPs have been grouped in different families, using sequence alignments [2–4] PolA, PolB, PolC, PolD, PolX, and PolY and reverse transcriptases. The main replicative DNAPs from Eukarya are found in family B, Bacteria in family C, and Archaea in families B and D. In recent years, a wealth of structural information brought to light the molecular mechanisms evolved by B- and C-family replicative DNAPs to fulfill the processivity and fidelity requirements for copying large genomes [5–7]. Such structural information is missing for D-family DNAPs (PolD), which is by far the least characterized DNAP at the structural level.

PolD is a heterodimeric replicative DNAP composed of a large catalytic subunit (DP2) and a smaller subunit with 3′-5′ proofreading exonuclease activity (DP1) [8–9]. Based on biochemical evidence, it has been proposed that PolD may act soon after initiation by the primase [10] and that at a later stage, a switch occurs such that PolB becomes responsible for leading strand replication while PolD continues to process the lagging strand [11]. PolD has been shown to be essential for cell viability [12–13] and is widely distributed among Archaea, being present in all four major superphyla: Euryarchaeota (including the methanogenic human symbionts); Diapherotrites, Parvarchaeota, Aenigmarchaeota, Nanoarchaeota, Nanohaloarchaeota (DPANN); the emerging Asgard superphylum; and Thaumarchaeota, Aigarchaeota, Crenarchaeota, Korarchaeota (TACK), only absent from Crenarchaeota [14]. Recently, we reported the individual crystal structures of the DP1 and DP2 catalytic cores, thereby revealing that PolD is an atypical DNAP that has all functional properties of a replicative DNAP but with the catalytic core of an RNA polymerase (RNAP) [15]. Indeed, PolD has been shown to share an unexpected structural homology with the “two-double-psi β-barrel” (DPBB) family of RNAPs [16], which includes multisubunit transcriptases from all domains of life, homodimeric RNA silencing pathway RNAPs, and atypical RNAPs encoded by some viruses [17]. All these nucleotide polymerases share a common catalytic center that is formed between two DPBBs, which contribute distinct amino acid residues to the active site in an asymmetrical fashion [16, 18–20].

However, DP1 and DP2 crystal structures were obtained separately and do not provide a comprehensive description of the molecular mechanisms of DNA polymerization and proofreading by PolD. Furthermore, in the DP2 (1–1050) crystal structure, the active site was flexible and partly unresolved in the electron density, and a critical C-terminal domain (CTD) of DP2 (1051–1270), which participates in the association with DP1, was deleted for crystallization purposes.

Here, we report the cryo–electron microscopy (cryo-EM) structure of the DNA-bound PolD complex. This structure sheds light on DNA-binding domains evolved by PolD to perform DNA replication and extends the repertoire of protein domains known to be involved in DNA replication. This work also enables a detailed comparison of PolD both with the cellular transcriptases and the eukaryotic replicative DNAPs.

## Results

### Structure determination of the EM density-based atomic model of PolD

**Fig 1:**
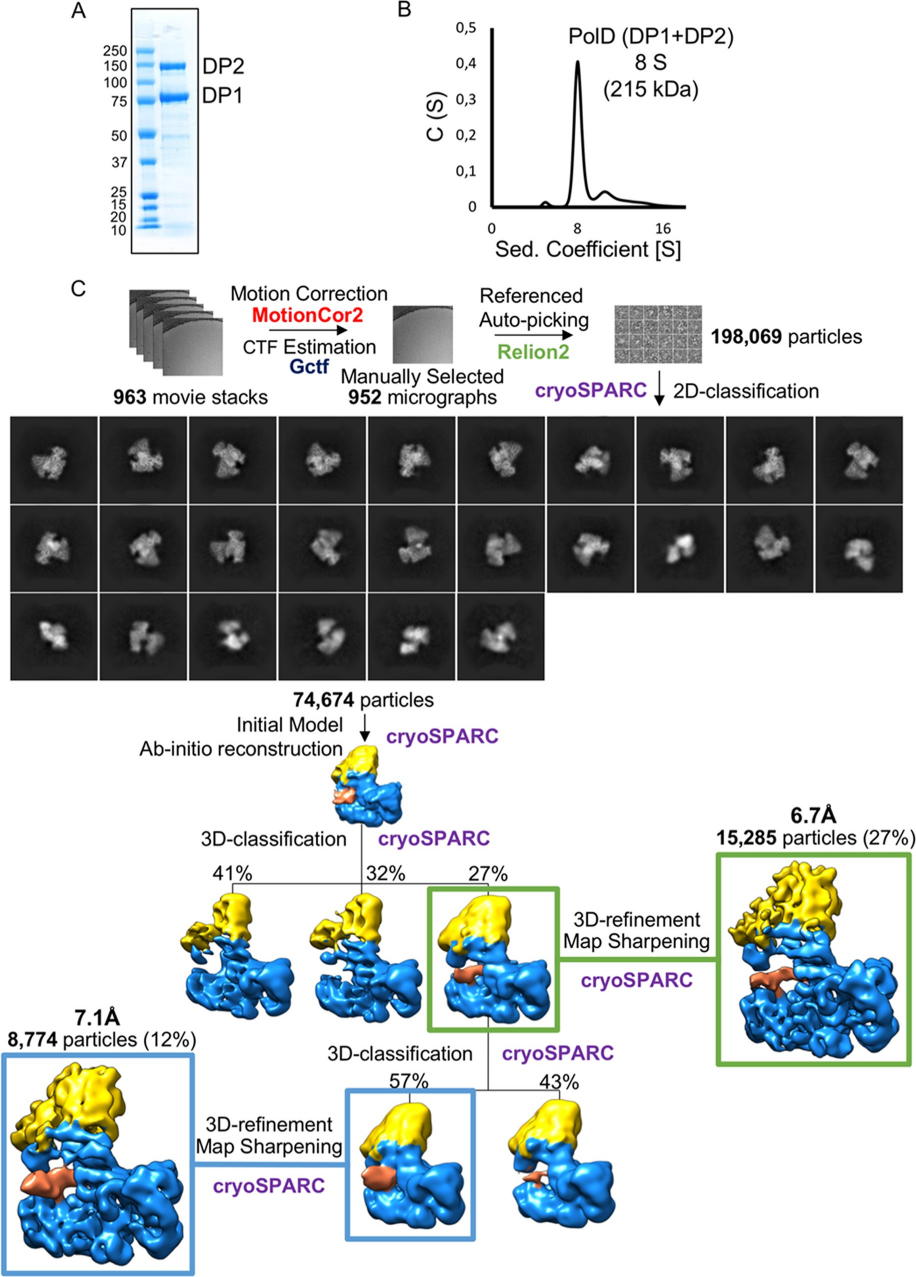
Microscopy data analysis of the heterodimeric DP1–DP2 complex of PolD bound with DNA. (A) SDS-PAGE analysis of purified PolD. DP1 and DP2 denote bands with an apparent molecular weight of about 75 kDa and 140 kDa, respectively. (B) Analytical ultracentrifugation sedimentation velocity analysis of PolD. PolD sediments at 8 S with a frictional ratio of 1.4 compatible with a 1:1 heterodimer composed of DP1 and DP2. (C) The image-processing workflow is shown for motion correction, CTF estimation, and autopicking in RELION-2.1.0 [22], as well as for 2D classification (selected 2D class averages are indicated), ab initio initial model reconstruction, 3D classification, refinement, and map sharpening in CryoSPARC-0.6.5 [23]. DP1 and DP2 volumes are respectively represented in yellow and blue. The DNA duplex volume is represented in coral. CTF, contrast transfer function.

The *Pyrococcus abyssi* PolD complex (215 kDa) consists of two subunits, DP1 (75 kDa) and DP2 (140 kDa), and could be coexpressed and copurified to homogeneity (Fig 1A). PolD behaves as a 1:1 heterodimer in analytical ultracentrifugation (Fig 1B). The PolD exonuclease-deficient variant [21] (DP1 H451A) was incubated with a 15-mer primed DNA duplex with a 5-nt overhang, mimicking DNA replication intermediates, to prepare samples for cryo-EM. After four rounds of 2D classifications, an overall dataset of 74,674 particles was obtained from 952 micrographs. Ab initio reconstruction of PolD is arranged in two lobes, intimately associated, that are compatible with the small DP1 and large DP2 subunits. Although no structurally distinct groups of PolD emerged from subsequent 3D classifications, the density corresponding to DNA is unequally well resolved from one subset of particles to the other, thereby suggesting partial occupancy and/or some flexibility of the DNA. The best electron density was obtained from a subset of 8,774 particles isolated from iterative 3D classifications and yielding to a density map at an overall resolution of 7.1 Å (Fig 1C). This 8,774-particle subset emerged from a previous 3D class (15,282 particles) that could be refined to 6.7 Å. Although this model displays a slightly higher resolution, the density corresponding to DNA is weaker, so it will not be discussed further.

**Fig 2:**
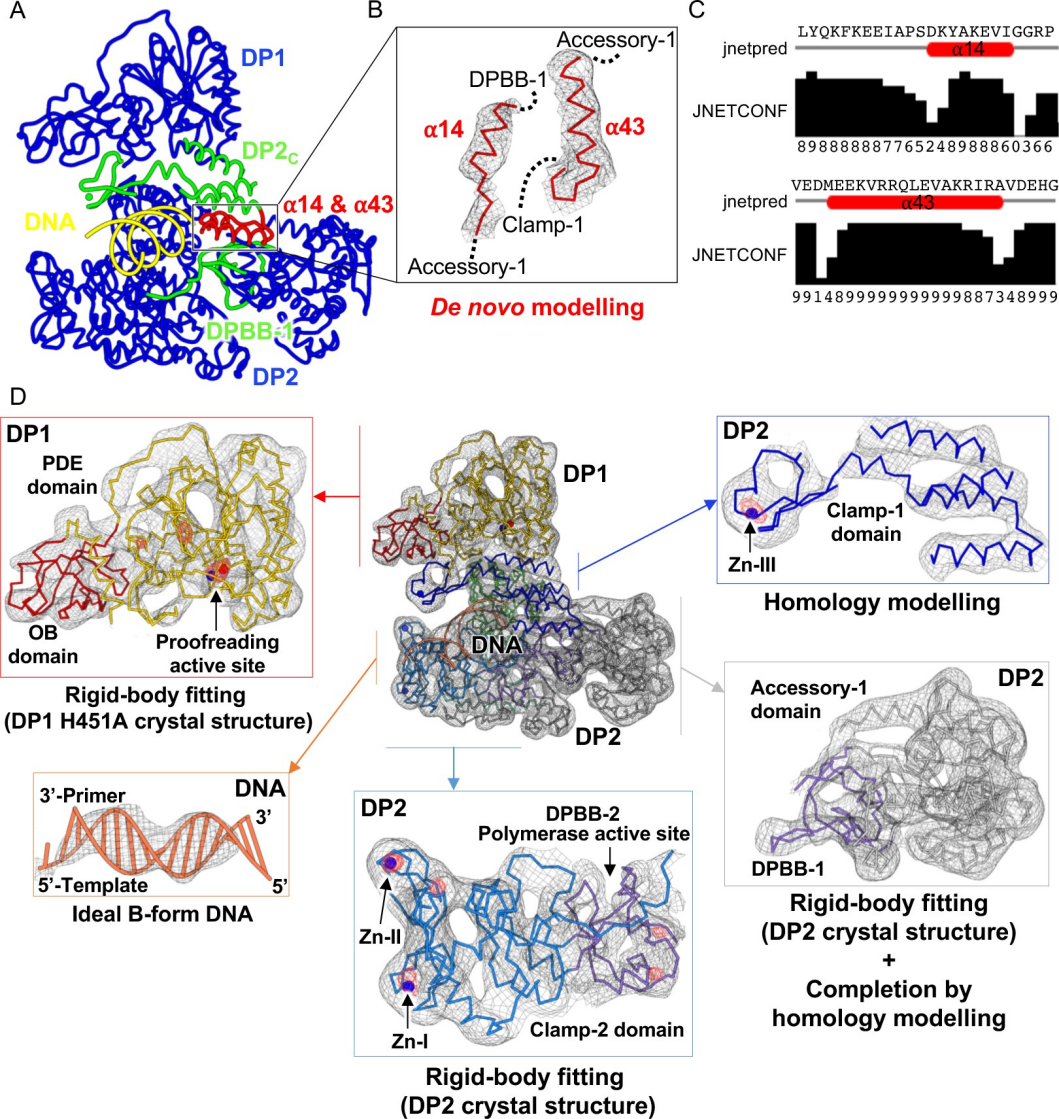
Cryo-EM reconstruction of the DNA-bound PolD binary complex. (A) Backbone trace of the cryo-EM model of PolD. The blue region was built by fitting the DP1 H451A (PDB ID: 6HMF, this study) and DP2 (PDB ID: 5IJL [15]) crystal structures in the cryo-EM map. The C-terminal region of DP2 (1090–1195) (green) was built guided by the homology with the CTD of the catalytic subunit of human Polε (PDB ID: 5VBN [24]) and adjusted manually in the cryo-EM density. Part of the DPBB-1 domain and the DPBB-connecting loop (green) were built by homology modeling guided against the structure of yeast RNAP-II (PDB ID: 2E2I [25]). A 15-/16-mer B-form DNA was generated with Coot [26] (yellow) and rigid-body fitted into the cryo-EM density. Finally, two α helices (red) were built de novo in the cryo-EM density: α14 (324–338) and α43 (1074–1089)). (B) Enlarged view showing the α14 and α43 α helices that were built de novo. The cryo-EM map surrounding these helices is shown as a gray mesh contoured at 6 σ. (C) Secondary structure predictions of Jpred [27] for the de novo–built α14 and α43 α helices. The confidence factor is indicated: 0, not confident, and 9, very confident. (D) Detailed views of the DNA-bound PolD cryo-EM experimental map. Density surrounding PolD and DNA is shown in gray mesh contoured at 6 σ. Residual peaks of density are shown in red mesh contoured at 12 σ. cryo-EM, cryo–electron microscopy; CTD, C-terminal domain; DPBB, double-psi β-barrel; OB, oligonucleotide binding; PDB, Protein Data Bank; PDE, phosphodiesterase domain; RNAP, RNA polymerase.

**Fig 3:**
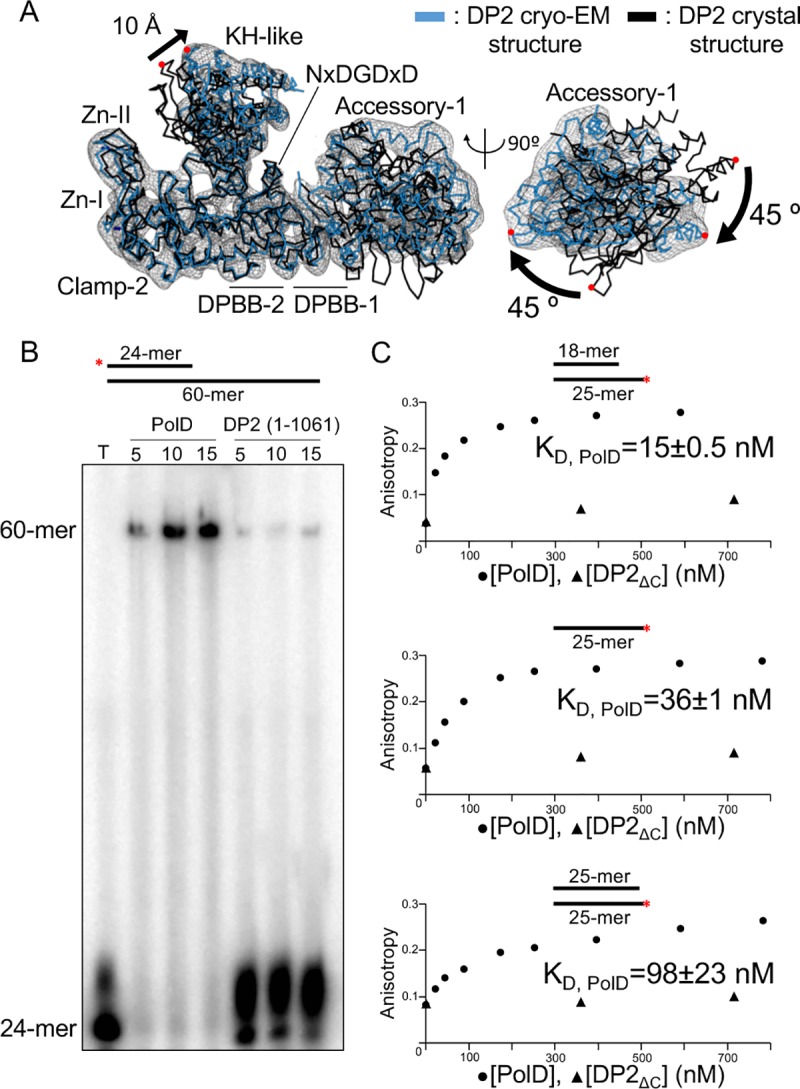
The DP1–DP2 heterodimeric assembly is required for PolD activity. (A) Movements of the DPBB-1, KH-like, and accessory-1 domains observed between the DP2 (1–1061) crystal structure (PDB ID: 5IJL [15]) and the PolD cryo-EM structure, supported by the cryo-EM density contoured at 6 σ (gray). Movements are indicated by arrows. Backbone traces of the DP2 cryo-EM structure and the DP2 (1–1061) crystal structure are shown in blue and black, respectively. (B) Primer extension activities of the heterodimeric DP1–DP2 PolD complex and the DP2 (1–1061) construct. A 5′-^32^P-labeled 24-mer (red asterisk) associated with a 60-mer template was used as a substrate. A control experiment was performed in absence of polymerase (lane T). (C) DNA-binding abilities of the heterodimeric complex of PolD and the DP2 (1–1061) construct. The two constructs were added to hexachlorofluorescein-labeled primer/templates, ssDNA substrates, and dsDNA substrates, and the increase in fluorescence anisotropy was noted. The underlying data can be found in S1 Data. K_D_ values (average ± standard deviation from three determinations) for the binding of PolD were determined as detailed in S1 Text. cryo-EM, cryo–electron microscopy; DPBB, double-psi β-barrel; dsDNA, double-stranded DNA; KH, K-homology; PDB, Protein Data Bank; ssDNA, single-stranded DNA.

The quality of the map is confirmed by the visualization of individual helices and ß sheets (Fig 2). Distribution of the local resolution shows that the most resolved region of the PolD–DNA complex map (approximately 5-Å resolution) are the exonuclease and polymerase catalytic cores of the DP1 and DP2 subunits (S1 Fig). Most of the cryo-EM maps can be interpreted by fitting the high-resolution crystal structures of the PolD DP1 (144–619) and DP2 (1–1050) individual subunits [15] (Fig 2). In order to prevent DNA degradation during cryo-EM preparation, the exonuclease-deficient variant [21] PolD (DP1 H451A) was used in this experiment. The structure of the DP1 H451A (144–619) individual subunit was solved separately using X-ray crystallography at 2.6-Å resolution, ensuring that neither the overall structure of DP1 nor its two metal ion–binding sites are altered by the H451A mutation (S2 Fig). All the density accounting for DP1 could be conveniently interpreted by rigid-body fitting the DP1 H451A individual crystal structure, with the DP1 EM and crystal structures showing only little differences. On the other hand, large interdomain rearrangements are observed between the DP2 crystal and cryo-EM PolD structures (Fig 3A). The DP2 crystal structure was split into separate domains and subdomains, which were fitted using real-space rigid-body refinement. Some regions in the active site of DP2 (48 residues) and the DP1–DP2 interacting region (91 residues) were built guided by the homology with RNAPs and the CTD of the catalytic subunit of Polε [24], respectively (Fig 2A). A region corresponding to two α helices (33 residues) was traced de novo according to secondary structure predictions (Fig 2B and 2C). Residual density remaining at high-level contouring is colocalized with the three zinc-binding domains contained in DP2 and the binuclear Fe/Zn catalytic center of DP1, thereby assessing the quality of the map and supporting the agreement between the map and the fitted model (Fig 2D). In the DNA-bound PolD structure, a 15-mer/16-mer primer/template duplex B-form DNA was docked in the cryo-EM density, guided by the unambiguous density for the duplex region, showing minor and major grooves (Fig 2D). However, no clear density is observed for the four 5′-terminal nucleotides of the template single-stranded DNA. Side chains deduced from existing crystals structures, including all side chains of DP1 and most side chains of DP2 (86%), were included in the model used for real-space refinement in Phenix. However, as the full model for PolD was built into a cryo-EM density map at intermediate resolution, we have deposited only Cα positions of PolD, DNA, and metal ions to ensure that coordinates are not overinterpreted.

### Architecture of the PolD–DNA-binding site suggests a structure-based nomenclature for D-family DNAPs

**Fig 4:**
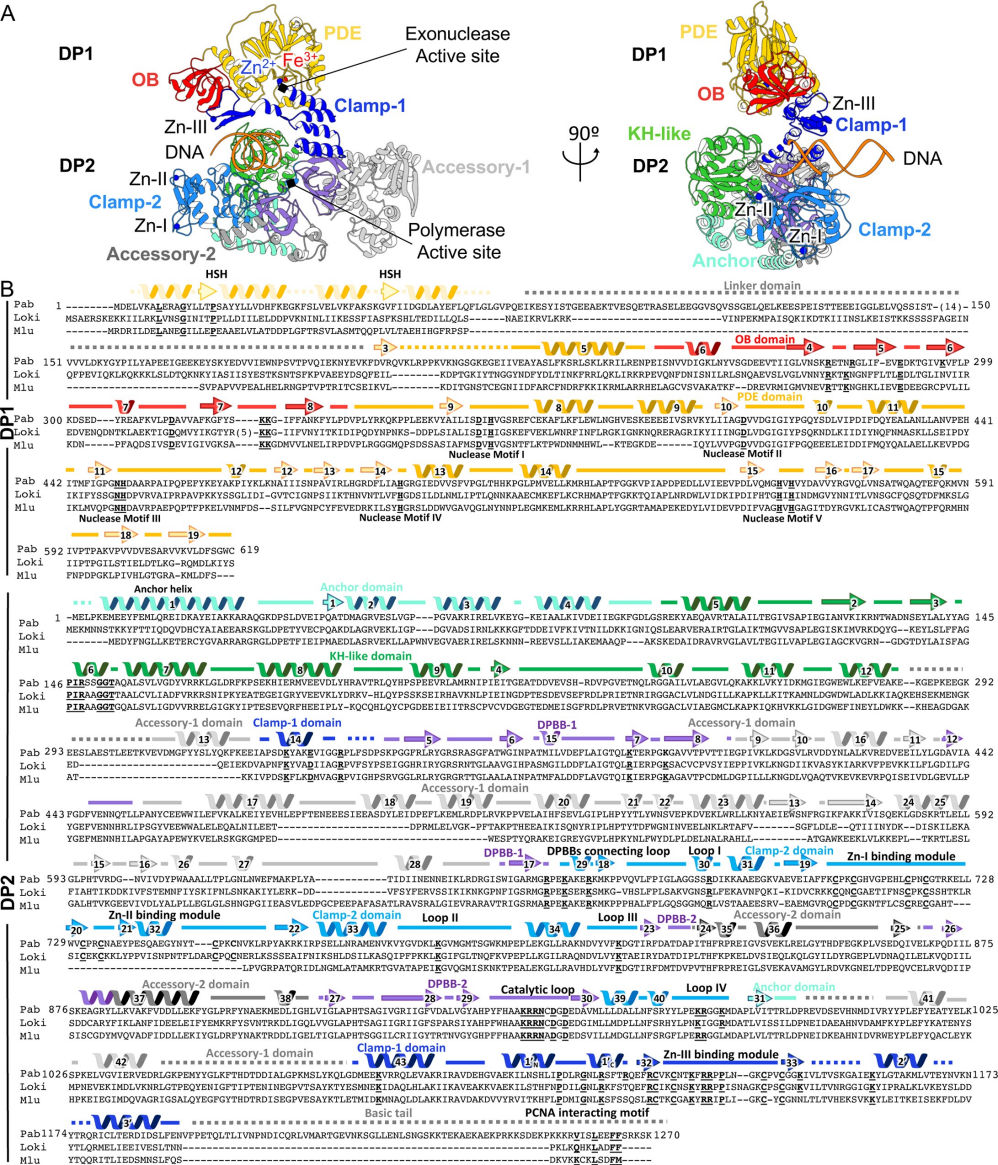
Structure of the PolD heterodimeric DP1–DP2 complex bound with DNA. (A) Ribbon diagram of the DNA-bound PolD structure highlighting the domains and domain-like regions that compose the DP1 and DP2 subunits. Zn^2+^ (blue) and Fe^3+^ (red) ions are shown as spheres. (B) Multiple-sequence alignment of the DP1 and DP2 subunits of PolD: *P*. *abyssi* (“Pab”) with *Lokiarchaeum* sp. *GC14_75* PolD (“Loki”) and *Methanomassiliicoccus luminyensis* PolD (“Mlu”). Secondary structure elements are colored according to panel (A). Functionally important conserved residues are highlighted. DPBB, double-psi β-barrel; HSH, helix-strand-helix; KH, K-homology; OB, oligonucleotide binding; PCNA, proliferating cell nuclear antigen; PDE, phosphodiesterase domain.

**Fig 5:**
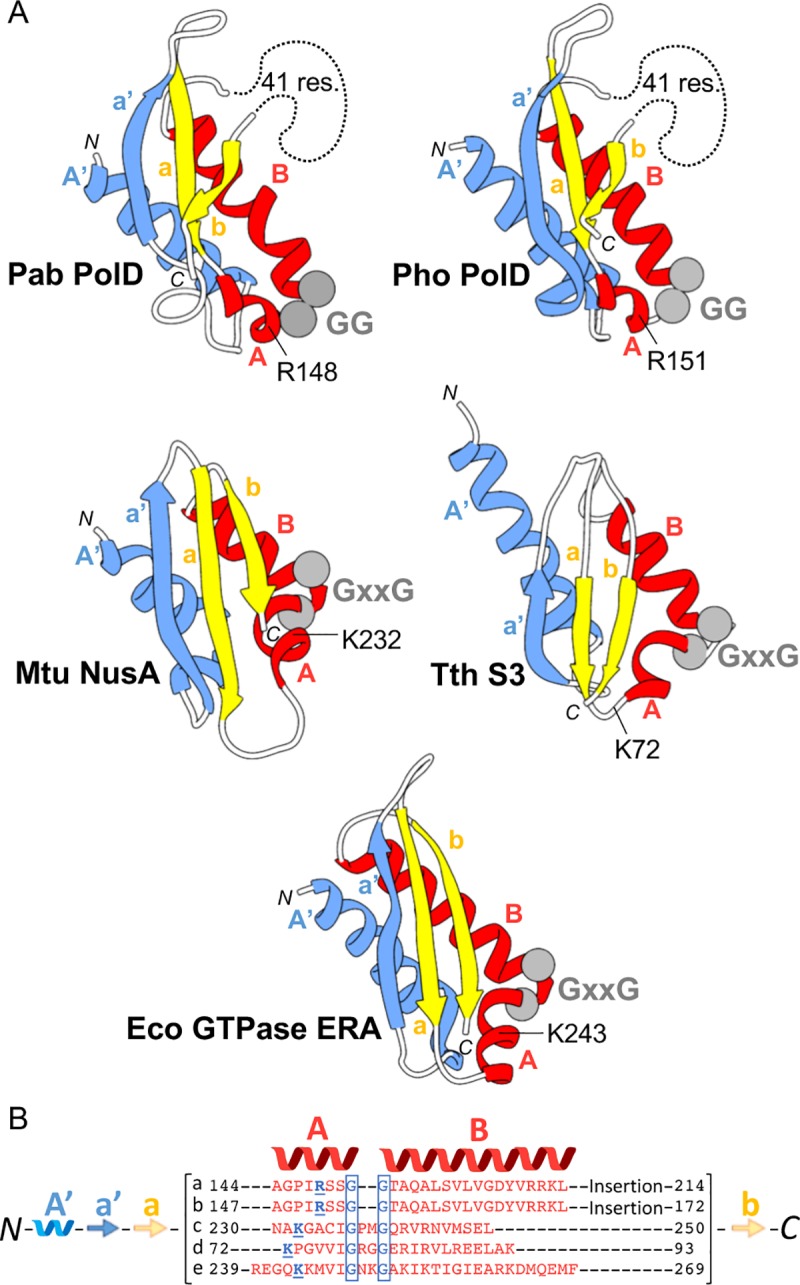
A KH-like domain orients the DNA template toward the active site of PolD. (A) Comparison of the PolD KH-like domain with type II KH domains: from top to bottom, the ribbon diagrams of KH domains from *P*. *abyssi* (“Pab”) DP2 (PDB ID: 5IJL [15]), *P*. *horikoshii* DP2 (PDB ID: 3O59 [28]), *Mycobacterium tuberculosis* (“Mtu”) NusA (PDB ID: 2ASB [33]), *Thermus thermophilus* (“Tth”) ribosomal protein S3 (PDB ID: 4OX9 [34]), and *E*. *coli* (“Eco”) GTPase ERA (PDB ID: 1EGA [31]). Cα atoms of conserved Gly residues are depicted with gray spheres. (B) Structure-based sequence alignment of the A and B helices from structures shown in (A). a: Pab PolD, b: Pho PolD, c: Mtu NusA, d: Tth S3, e: Eco GTPase ERA. Conserved Gly and basic residues (“res.”) are highlighted in blue. Gly, glycine; GTPase, guanosine triphosphatase; KH, K-homology; PDB Protein Data Bank.

The structure of the native DNA-bound PolD sheds light on critical DNA interaction domains, enabling us to propose a structure-based nomenclature for D-family DNAPs (Fig 4). PolD shows a claw-shaped active site, at the center of which is the two-DPBB catalytic core and at the edge of which are three zinc-binding modules named the Zn-I, Zn-II, and Zn-III domains. The DNA substrate is cradled between a bipartite clamp domain, named 1 and 2, emanating from DPBB-1 and DPBB-2, respectively. The clamp domain is barricaded from one side by a globular domain (85–283) located in the N-terminal region of DP2, which is ideally located to orient the DNA template in the active site. Consistently, former biochemical studies have shown that this domain binds DNA, with a marked preference for 3′-recessed DNA over double-stranded DNA (dsDNA) [28]. The DP2 N-terminal domain was compared to the Protein Data Bank (PDB) using Dali [29] and shown to share structural homology with several K-homology (KH) domains (highest z-score = 5.7) (Fig 5). Eukaryotic type I and archaeal/bacterial type II KH domains are ancestral single-stranded nucleic acid–binding folds that share a β-α-α-β minimal core motif and two additional α and β elements, which are either located in the C-terminal (type I) or the N-terminal (type II) region of the KH core motif [30]. The DP2 KH-like domain shares the topology of type II KH domains and can be superimposed onto the *Escherichia coli* guanosine triphosphatase (GTPase) ERA [31] over 80 Cα with a root-mean-square deviation (r.m.s.d.) of 3.6 Å. The most prominent sequence conservation among KH domains is a conserved GxxG motif that is located within a loop that links the two α helices of the KH minimal core and directly contacts nucleic acids. These two glycine residues are superposed and conserved with two canonical consecutive glycine residues (G151 and G152) of the DP2 KH-like domain. KH domains have already been predicted in the N-terminal region of bacterial PolC [32], and the cryo-EM structure of PolD shows—for the first time in a structural context, to our knowledge—a KH domain associated with a replicative DNAP. Two accessory domains, named -1 and -2 (emanating from insertions in DPBB-1 and DPBB-2 subdomains, respectively), play a structural role by scaffolding together the essential two-DPBB catalytic cores and the clamp-1 and clamp-2 domains. The KH-like domain is connected to the anchor domain, which firmly attaches it to the clamp-2 domain and orients it in the active site (Fig 4).

A characteristic of PolD is that the exonuclease and polymerase active sites are hosted by two distinct subunits. DP1 shows an oligonucleotide binding (OB) domain that is inserted within the N-terminal region of a large Mre11-like nuclease phosphodiesterase domain (PDE) whose active site entry faces the 3′ end of the nascent DNA strand. DP1 interacts extensively with the C-terminal region of DP2, which is part of clamp-1. Comparison of the cryo-EM PolD structure with the DP2 (1–1061) crystal structure reveals that the association with DP1 is required for rearranging the DP2 catalytic core into an active conformation. Indeed, in the DP2 (1–1061) crystal structure, deletion of the CTD of DP2 causes a profound reorganization of the active site (Fig 3A): (1) the DPBB-1 domain is partly disordered and moves with respect to DPBB-2 from the canonical relative orientation of the two-DPBB catalytic center that is shared within all cellular transcriptases and full-length PolD, (2) clamp-1 and the accessory-1 domains are rotated by about 45° with respect to clamp-2, and (3) the KH-like domain moves by about 10 Å away from the catalytic center. Consistently, in a DNA-elongation activity assay, the native DP1–DP2 complex shows a far higher activity than the DP2 (1–1061) catalytic core on its own (Fig 3B). Altogether, these data show that association with DP1 is required to rearrange the catalytic core of DP2 in an active conformation.

### Structural basis for the association between the DP1 and DP2 subunits of PolD

**Fig 6:**
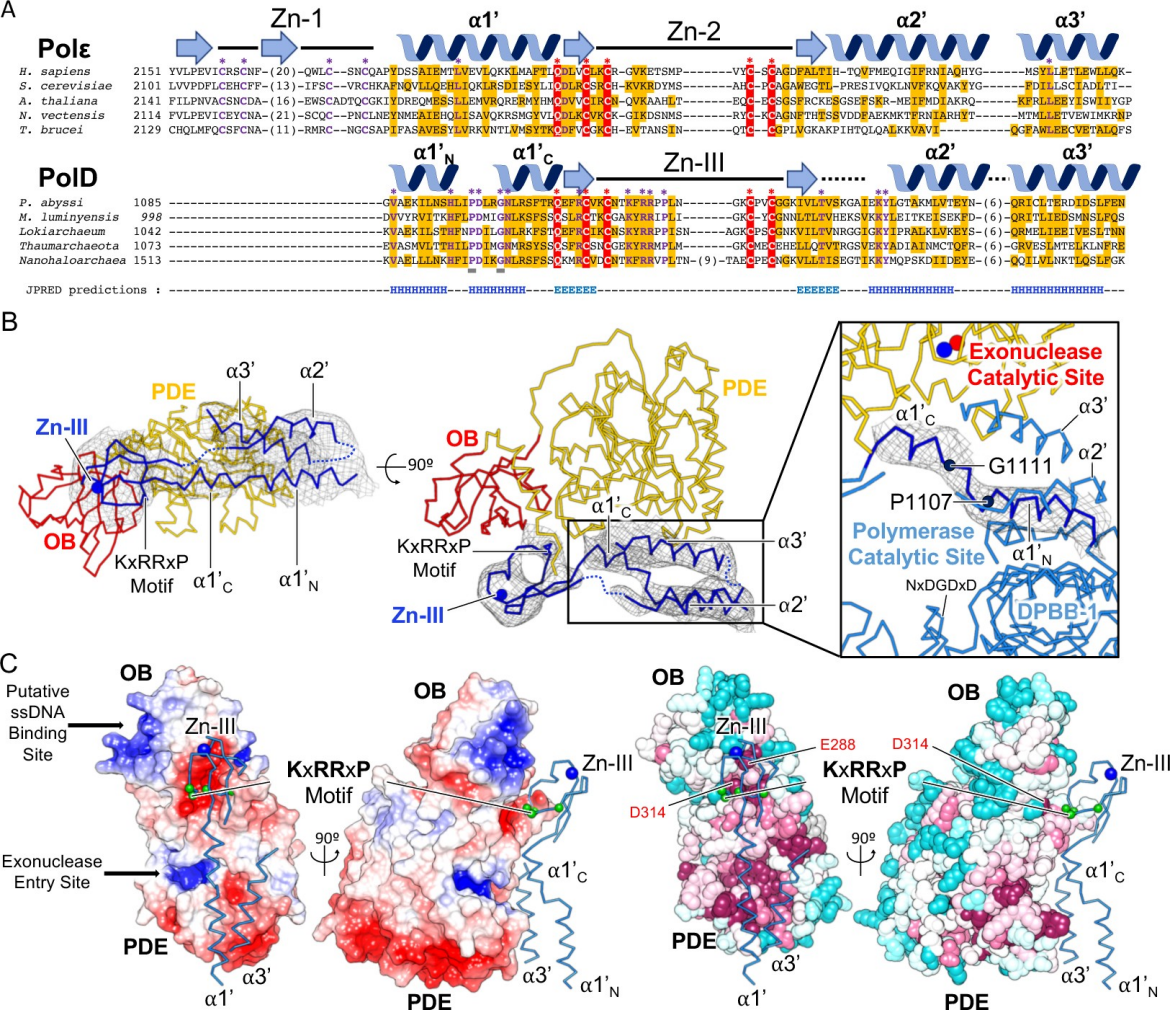
Structural basis for the interaction between DP1 and DP2. (A) Structure-based sequence alignment of the C-terminal regions of *Homo sapiens* Polε A subunit (PDB ID: 5VBN [24]) and *P*. *abyssi* PolD DP2. Secondary structure elements are shown above the sequence alignment according to the crystal structure of the *H*. *sapiens* Polε and the cryo-EM structure of PolD. Sequence similarity is highlighted with yellow boxes, and conserved residues are highlighted with red boxes. The “helix-breaker” motif is indicated with gray lines. Two connecting loops (1149–1155) and (1171–1777) were omitted because of the lack of density. Secondary structures predictions from Jpred [27] of the *P*. *abyssi* PolD DP2–CTD are shown below the sequence alignment. (B) Structure of the DP1/DP2-CTD interface. Density surrounding the DP2–CTD is shown in gray mesh and contoured at 6 σ. Disordered loops are indicated by dotted blue lines. The right panel shows an enlarged view of the cryo-EM density that surrounds the α1’_N_ and α1’_C_ helices. (C) Overall view highlighting the contribution of the DP2_C_ helices α1’ and α3’ and the Zn-III binding domain to the DP1–DP2 interface. DP2–CTD is shown as ribbon, and the Zn^2+^ ion is represented as a blue sphere. Cα atoms of the KxRRxP motif contained in the Zn-III binding domain are shown as green spheres. Left: Charge distribution at the interface of the DP1–DP2 complex. Electrostatic surface potentials are generated using the DP1 H451A crystal structure (PDB ID: 6HMF, this study) fitted into the cryo-EM density. Right: Same view showing the evolutionary conservation of the surface amino acids mapped on DP1, calculated with Consurf [37] from an alignment of 150 DP1 sequences. Amino acid conservation is indicated by a transition in color hues, from magenta (most conserved) to cyan (most variable). cryo-EM, cryo–electron microscopy; CTD, C-terminal domain; DPBB, double-psi β-barrel; OB, oligonucleotide binding; PDB, Protein Data Bank; PDE, phosphodiesterase domain; ssDNA, single-stranded DNA.

Prior to this work, the DP1–DP2 interacting region had never been modeled. This region was deleted from our previous constructs in order to crystallize the DP2 catalytic domain [15]. Former bioinformatics studies showed that the C-terminal region of DP2 hosts a zinc-binding domain resembling that of the eukaryotic replicative Polε [35] (Fig 6A). Taking advantage of the recent crystal structure of the human Polε B subunit in complex with the CTD of its catalytic subunit [24], a homology model (18% identity / 48% similarity) covering residues 1096–1195 of DP2 was calculated using Phyre [36]. The DP1–DP2 interfacial region shows electron density that can be accounted for by a bundle of four α helices and a globular region that resembles a zinc-binding domain. The homology model can be conveniently fitted in the electron density, guided by the clear density for the helical bundle and by the globular density corresponding to the Zn-III domain (Figs 2D and 6B). The model was modified manually to better account for the density. In particular, the first α helix (named α1’) splits into two α helices, named α1’_N_ and α1’_C_. Consistent with secondary structure predictions, the helix is broken at the level of two canonically conserved proline and glycine residues (P1107 and G1111, respectively) (Fig 6A and 6B). The helix α1’_N_ contributes to fold the α1’-3′-helical bundle and interacts with the rest of the DPBB-1 catalytic core, whereas the helix α1’_C_ interacts with DP1 (Fig 6B).

The DP1 OB–PDE interface forms an electronegative groove interacting intimately with a loop carried by the Zn-III domain, which hosts canonical basic residues (R1131 and R1132) (Fig 6C). The key location of the DP2 Zn-III domain in the DP1-DP2 interaction area rationalizes former studies demonstrating that a 20-amino-acid-long peptide covering the Zn-III domain shows nanomolar affinity with DP1 in *P*. *horikoshii* PolD [38]. In addition, helices α1’_C_ and α3’ of DP2 interact with loops emanating from the core secondary elements of the PDE (Fig 6C). Consistently, the DP1–DP2 interacting crevice shows the highest degree of evolutionary conservation relative to the rest of the solvent-exposed regions of the DP1 surface (Fig 6C). Our model thus suggests that a minimal core for the interaction between DP1 and DP2 would involve the 1096–1195 DP2–CTD region and a large region of DP1 covering both OB and PDE (144–619). Consistently, DP1 (144–619) fused to a His-Tag comigrated with DP2 (1096–1195) in chromatographic affinity column, and the complex could be copurified through a three-chromatographic-steps protocol, including a step with salt concentration up to 1 M NaCl, suggesting that this interaction is highly specific and stable (S3 Fig). Attempts to express DP2–CTD (1096–1195) or DP2 full-length invariably resulted in aggregation, showing that DP1–DP2 forms a constitutive complex and that association with DP1 is required to stabilize the DP2–CTD. Similar results have been reported for PolD from *P*. *horikoshii* [38–39].

### Structural basis for DNA binding by PolD as revealed at medium resolution

All replicative DNAPs have evolved protein domains named palm, fingers, and thumb domains arranged to form the DNA-binding cleft. The palm domain carries the catalytic residues, the fingers domain drapes over the nascent base pair, and the thumb domain holds the DNA duplex during replication and contributes to processivity [40–41]. Equivalent DNA-binding domains could not be predicted in PolD from the individual crystal structures of the DP1 and DP2 subunits [15]. Although the reported DNA-bound cryo-EM structure of PolD solved at an intermediate resolution does not allow for a detailed description of the polymerization mechanism by PolD, it reveals—for the first time, to our knowledge—the DNA-binding site of PolD, extending the repertoire of domains known to be involved in DNA replication.

**Fig 7:**
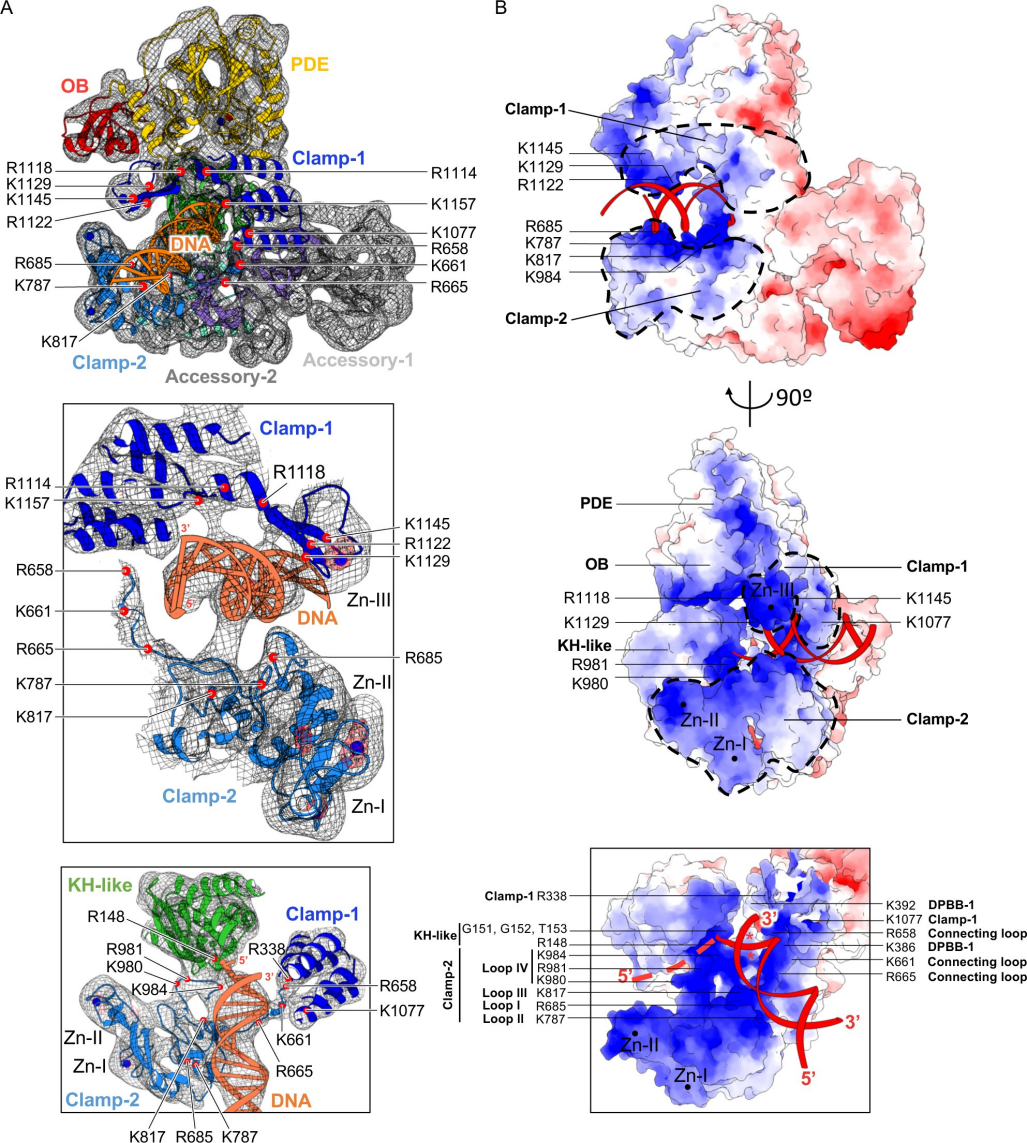
Structure and surface charge distribution of the PolD–DNA-binding site. (A) Selected views of the DNA-bound PolD cryo-EM experimental map. Upper: Overview of the DNA-bound complex. Middle: The minor groove of the nascent DNA duplex is cradled between clamp-1 and clamp-2 domains. Lower: Enlarged view showing the contributions of clamp-1, clamp-2, and the KH-like domain to the PolD–DNA-binding site. For clarity, the Zn-III binding domain is hidden. Density surrounding PolD and DNA is shown as a gray mesh contoured at 6 σ. Residual peaks of density are shown in red mesh contoured at 12 σ. Cα of conserved basic residues are shown as red spheres. (B) Surface charge distribution of the PolD–DNA-binding site. The surface of PolD is colored according to the electrostatic surface potential, with negative, neutral, and positive charges shown in red, white, and blue, respectively. The electrostatic surface potential was calculated using a PolD model containing all side-chain atoms. All side chains of DP1 and most side chains of DP2 (86%) were deduced from the existing crystal structures; the others were modeled according to the most likely rotamers. Catalytic aspartic residues are marked by a red asterisk. Zn-binding domains are depicted with black spheres. Clamp-1 and clamp-2 domains are highlighted by dotted lines. The DNA backbone is colored red, and the last 4 nt of the 5′ template are schematized with red dashes, based on the position of the KH-like domain. cryo-EM, cryo–electron microscopy; DPBB, double-psi β-barrel; KH, K-homology; OB, oligonucleotide binding; PDE, phosphodiesterase domain.

PolD has evolved a specific bipartite clamp domain, which completely surrounds the DNA duplex (Fig 7). Clamp-1 and clamp-2 domains contribute a central cleft with a diameter of 30 Å, which is located upstream of the DP2 polymerase catalytic center. Clamp-1, which consists of a 5-helix bundle and the Zn-III domain, faces from one side the polymerase catalytic center and from the other side the nuclease catalytic center (Figs 4A and 7). Clamp-1 contributes several canonical basic residues (R1122, K1125, K1129, and K1145), which form a highly biased distribution of positively charged side chains and contribute to surround the nascent DNA duplex. In addition, Zn-III domain, as well as the β strands connecting Zn-III domain to the clamp-1 helical bundle, is ideally located to interact with the minor groove of the nascent duplex DNA (Fig 7). The critical role of clamp-1 in DNA replication suggested by the cryo-EM structure is supported by a functional study showing that substituting two cysteines of the Zn-III domain severely impairs the activity of PolD from *Archaeoglobus fulgidus* [42]. PolD shares with its RNAP counterparts the N-terminal helix of the 5-helix bundle, which is part of a hybrid-binding domain in two-DPBB RNAPs. This helix contains two canonical basic residues and shares a similar orientation with respect to the two-DPBB catalytic core and the nascent duplex in both families of enzymes [25]. Clamp-2 encompasses the region between the C terminus of the last β strand of DPBB-1 and the first β strand of DPBB-2, as well as the C-terminal extension located at the C terminus of DPBB-2. It is mainly composed of four loops, named loop-I, loop-II, and loop-III—which form the second electropositive patch surrounding the neo-formed DNA duplex—and loop-IV, which is ideally located for binding the upstream DNA template (Fig 7B). Zn-I domain is located at the edge of clamp-2 and contributes to stabilizing clamp-2, which is otherwise composed mainly of loops. Zn-II domain is not strictly conserved among PolD polymerases and is inserted in the Zn-I domain (Fig 4B). In total, the Zn-III domain of clamp-1; loop-I, loop-II, and loop-III of clamp-2; and several residues of the two-DPBB catalytic center contribute conserved basic residues that form a circular clamp that binds about one turn of helix (10–12 base pairs) of the nascent DNA duplex (Fig 7). The role of this DNA-binding clamp was evaluated by comparing the DNA-binding properties of the heterodimeric PolD complex with the DP2 (1–1065) construct, whose clamp-1 domain is deleted (Fig 3C). As expected for a replicative DNAP, PolD binds primer-template DNA with a dissociation constant in the nanomolar range (15 nM). Similar values of DNA-binding constants have been reported previously for PolD from *P*. *furiosus* [43] and other replicative DNAPs [44]. The affinities of PolD for dsDNA and single-stranded DNA (ssDNA) were respectively 6.5-fold and 2.4-fold weaker, showing that PolD preferentially binds primed DNA. This observation is consistent with former gel-shift assays [11]. In contrast, the DNA-binding affinity of DP2 (1–1065) is strongly impaired compared to the entire PolD complex, thereby showing that the clamp binding domain is crucial for DNA binding by PolD (Fig 3C).

All DNA-bound structures of replicative DNAPs show that the single‐stranded part of the DNA template is flipped out of stacking arrangement with the duplex by a sharp angle in the template sugar-phosphate backbone [40]. Consistently, the DNA template enters the polymerase active site through a crevice that points toward the two-DPBB catalytic core and is orthogonal to the axis of the nascent DNA duplex. The putative DNA template entry site is lined by the OB domain of DP1, the Zn-II domain, clamp-2, and the KH-like DNA-binding domain. This region is particularly rich in basic residues that contribute a positively charged surface electrostatic potential that is very favorable for DNA binding (Fig 7B). In particular, the KH-like canonical motif (PIRxxGGT) (Fig 5) is located next to the first single-stranded nucleotide of the template strand (Fig 7). In addition, loop-IV of the clamp-II domain lines the putative DNA template-binding region and contributes several highly conserved basic residues, which form a positively charged surface that is ideally located to interact with the phosphate backbone of the DNA template (Fig 7B).

**Fig 8:**
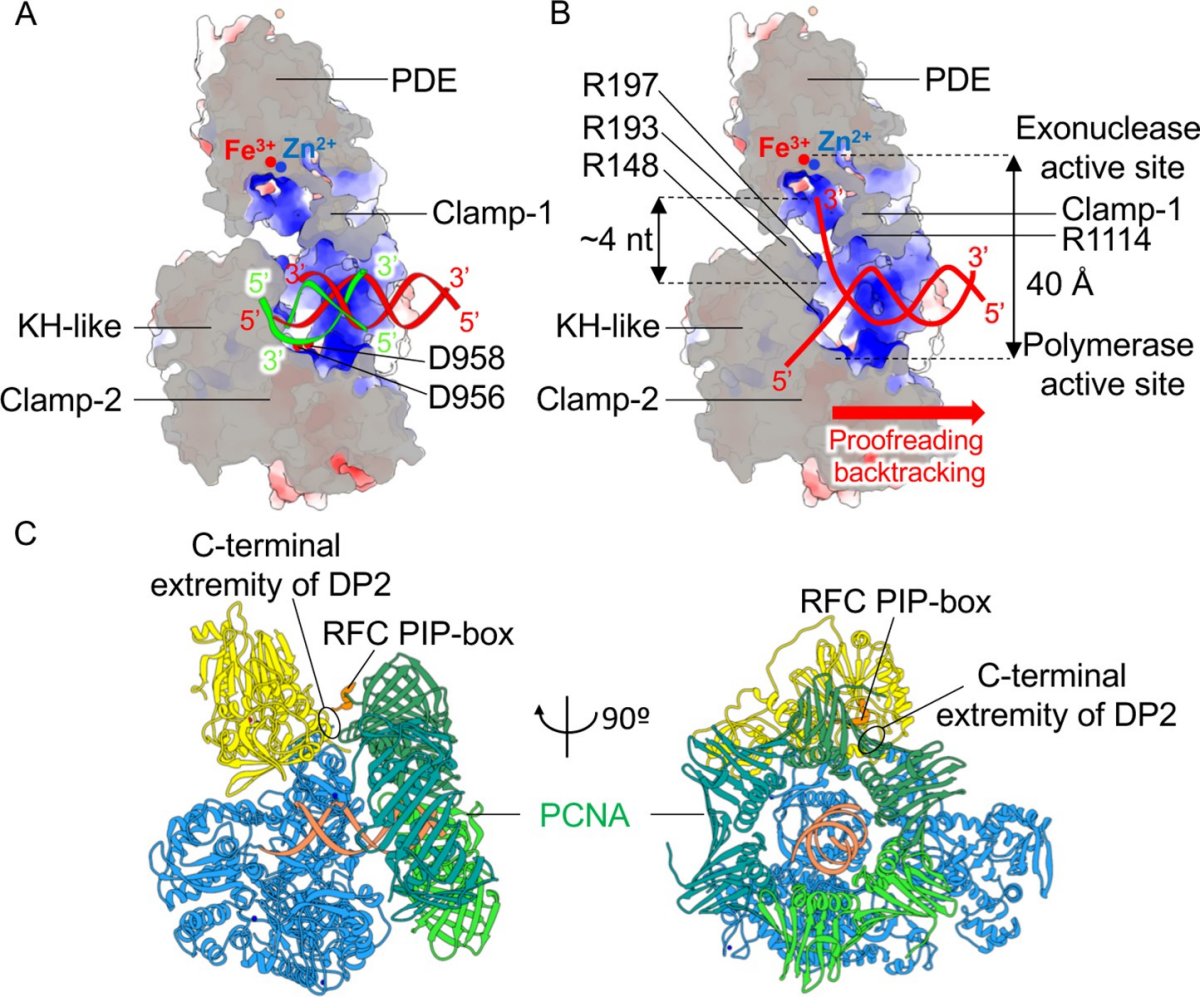
Proposed path for the 3′ end of the DNA primer during elongation and proofreading by PolD. (A) Cutaway front view of PolD showing a comparison of the position of DNA in the cryo-EM structure of PolD (red) and the nascent RNA/DNA hybrid (green) in the crystal structure of the elongation complex of *S*. *cerevisiae* RNAP-II [25]. The section plane crossing PolD is represented with gray area. The structures of PolD and RNAP-II are superimposed on their two-DPBB catalytic cores, showing that the nascent DNA duplex in PolD (red) and the nascent RNA/DNA hybrid in RNAP-II (green) share a similar orientation with respect to their two-DPBB catalytic core. However, in the structure of PolD, the DP2 KH domain prevents the DNA substrate from coming closer to the DNAP elongation site. This observation suggests that in the elongation mode, the KH domain would move in order to allow the 3′ end of the primer to access the elongation site, similarly to the finger domain in other families of DNAPs. (B) Same view as in (A) showing a putative path for the DNA being digested. The helical axis of the DNA duplex is identical to that of the DNA-bound PolD cryo-EM structure. The 3′ end of the DNA primer is extended by approximately 4 nt toward the exonuclease catalytic site of DP1. A red arrow shows the direction of the hypothetical proofreading backtracking mode of PolD. (C) Model of the PolD–DNA–PCNA ternary complex. cryo-EM, cryo–electron microscopy; DNAP, DNA polymerase; DPBB, double-psi β-barrel; KH, K-homology; PCNA, proliferating cell nuclear antigen; PDE, phosphodiesterase domain; PIP, PCNA-interacting peptide; RFC, replication factor C; RNAP, RNA polymerase.

The position of DNA in the cryo-EM structure of PolD can be compared with the many structures of DNA-dependent RNAPs solved in complex with DNA. When the structures of PolD and the elongation complex of *Saccharomyces cerevisiae* RNAP-II are superimposed on their two-DPBB catalytic cores, the nascent DNA duplex in PolD and the nascent RNA/DNA hybrid in RNAP-II show a very similar orientation with respect to their two-DPBB catalytic core (Fig 8A). However, whereas the 3′ end of the RNA primer is only 6 Å distant from the Cα of the catalytic residue D481 in the structure of RNAP-II, the 3′ end of the DNA primer is located about 15 Å from the corresponding catalytic residue in PolD DP2 (D956). In the PolD–DNA-bound binary complex, the DP2 KH domain prevents the DNA substrate from coming closer to the DNAP elongation site. This observation suggests that in the elongation mode, the KH domain would move in order to allow the 3′ end of the primer to access the elongation site, similarly to the finger domain in other families of DNAPs. Comparing the structure of the KH domain in the DP2 individual crystal structure and the cryo-EM structure shows that this domain is mobile and moved by about 10 Å between both structures (Fig 3A).

### A straight path leads to the DP1 exonuclease active site

The DP1 nuclease and DP2 polymerase active sites sandwich the 3′ end of the nascent DNA strand and are about 40 Å distant from each other, a feature shared with other replicative DNAPs [41] (Fig 8B). Although the cryo-EM PolD structure shows no contacts between DP1 and the bound DNA, the exonuclease active site is suitably located to catch the 3′ end of the nascent DNA strand. The phosphate moiety of the 3′-terminal nucleotide of the primer lies at a distance of 25 Å away from the exonuclease active site of DP1, which could be accounted for by a 4-nt-long ssDNA, a value shared with other DNAPs with proofreading activity [41]. The structure suggests that the DNA being digested would access the DP1 nuclease active site through a path that faces the KH-like domain from one side and helix α1’_C_ of clamp-1 from the other side. Consistently, this path is lined by several basic residues that may stabilize DNA binding. DNAPs with proofreading activity are able to sense mismatches by contacting the minor groove of base pairs beyond the insertion site [45–46]. The PolD cryo-EM structure shows that the Zn-III domain from clamp-1 and loop-I from clamp-2 are intimately interacting with the minor groove of the nascent duplex DNA, suggesting that they may contribute to sense mismatches (Fig 7).

### Modeling of the PolD–PCNA–DNA ternary complex

Replicative DNAPs generally operate in association with a sliding clamp that encircles the DNA and greatly enhances processivity. PolD has been shown to require the DNA sliding clamp proliferating cell nuclear antigen (PCNA) for high processivity. The crystal structures of the *P*. *furiosus* PCNA (PDB ID: 1ISQ) [47] (89% identity with *P*. *abyssi* PCNA) bound with a PCNA-interacting peptide (PIP) [48], as well as the crystal structure of the *H*. *sapiens* PCNA bound with DNA [49], have already been reported, thus facilitating modeling of the PolD–DNA–PCNA ternary complex (Fig 8C). Taking advantage of these crystal structures, we propose a structural model of the interaction between PolD and PCNA, which is consistent with former studies showing that PolD binds PCNA through multiple sites located in DP1 and DP2, including a PIP motif that is hosted in the C terminus of DP2 [50]. Indeed, the model shows that PolD and PCNA may interact through a wide surface of interactions overlapping with both DP1 and DP2 subunits and that the C-terminal extremity of DP2, which hosts a PIP motif (Fig 4B), colocalizes with the PCNA PIP box (Fig 8C). The model rationalizes how PCNA enhances the processivity of PolD by perpetuating the interactions with the nascent DNA duplex when it exits the PolD clamp. Binding to PCNA would thus substantially increase the region of interaction between PolD and the nascent DNA duplex, thereby preventing the polymerase from falling off prematurely [51].

### Shared structural features of RNAPs and PolD active sites

**Fig 9:**
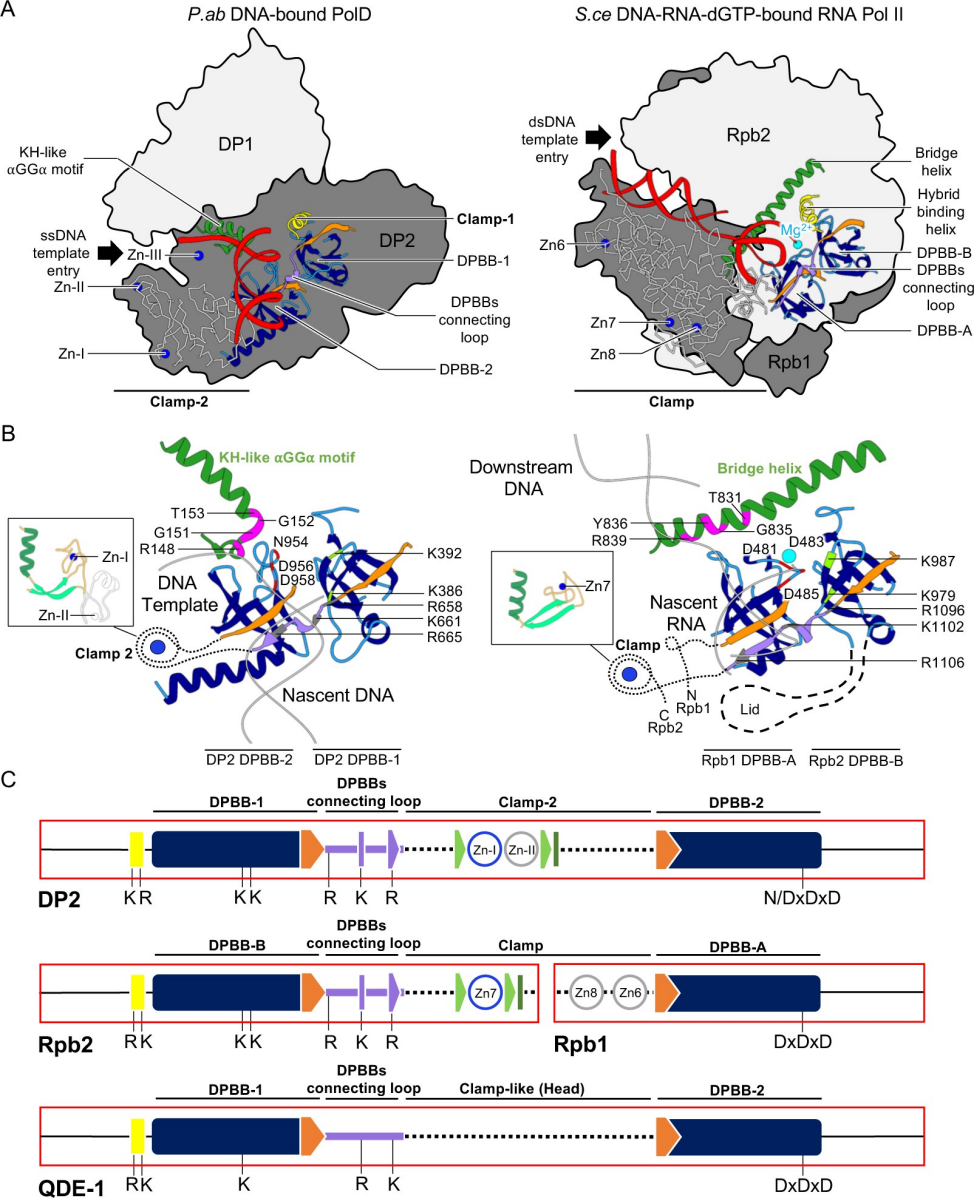
Structure-based comparison of PolD and two-DPBB RNAPs. (A) Shared structural elements between *P*. *abyssi* (“P.ab”) PolD and *S*. *cerevisiae* (“S.ce”) RNAP-II (PDB ID: 2E2I [25]). The structures of PolD and RNAP-II are aligned with respect to their two-DPBB catalytic core. Black arrows indicate the DNA template entry site in both PolD and RNAP-II active sites. DPBBs are colored in blue, with the last β strand of DPBB-1 (or DPBB-B) and the first β strand of DPBB-2 (or DPBB-A) colored orange. The conserved DPBB-connecting loop (purple) and template-binding helix (yellow) are also shown. The clamp-2 domain of PolD and the clamp domain of RNAP-II are depicted with a white backbone trace. The conserved αGGα motif of the PolD KH-like domain and the bridge helix of RNAP-II occupy the same location with respect to the nascent duplex. Zn-binding domains and Mg^2+^ ions are depicted with blue and cyan spheres, respectively. (B) Enlarged view of the shared structural elements between PolD and RNAP-II. (C) Genetic organization of these shared structural elements in PolD, DNA-dependent RNAPs, and QDE-1. dGTP, deoxyguanosine triphosphate; DPBB, double-psi β-barrel; dsDNA, double-stranded DNA; KH, K-homology; QDE-1, quelling defective phenotype; RNAP, RNA polymerase; ssDNA, single-stranded DNA.

The two-DPBB polymerase superfamily encompasses DNA-dependent RNAPs responsible for DNA transcription in all forms of cellular life, some viruses, and the eukaryotic RNA-dependent RNAPs (quelling defective phenotype [QDE-1]) involved in gene silencing [16, 52]. PolD substantially extends the two-DPBB superfamily to DNA-dependent DNAPs. Comparing the DNA-bound cryo-EM structure of PolD to the many structures of RNAPs enables us to delineate the minimal core of these DNA or RNAPs and discuss the molecular basis for their respective substrate specificities (Fig 9). Nevertheless, some conclusions cannot be extended to QDE-1, as its DNA-bound structure is not solved yet [52].

PolD and DNA-dependent RNAPs resemble a claw formed by the heterodimeric assembly of two subunits, DP1–DP2 for PolD and Rpb1–Rpb2 for *S*. *cerevisiae* RNAP-II [25] (Fig 9A and 9B). Both DNA-binding sites orient the DNA template entry and the nascent duplex exit in a same relative axis with respect to the two-DPBB catalytic core. The common architecture of their two-DPBB catalytic core is underpinned by the conservation of two invariant catalytic aspartic residues and two critical lysine residues, which have been shown to stabilize the 3′ end of the nascent RNA strand in DNA-dependent RNAPs (S4 Fig) [15]. Comparing the architecture of their DNA-binding sites extends the structural elements that are conserved among the two-DPBB superfamily beyond their canonical two DPBBs. First, PolD shares with its RNAPs counterparts an α helix that is part of a hybrid-binding domain in DNA-dependent RNAPs. This helix is connected to the N-terminal end of DPBB-1 (DPBB-B, in DNA-dependent RNAPs) and shares a similar orientation with respect to the two-DPBB catalytic core and the nascent duplex. This helix hosts two well-conserved basic residues (*P*. *abyssi* DP2 K329, R338 and *S*. *cerevisiae* Rpb2 R766, K775). The side chain of K775 interacts with the phosphate backbone of the nascent RNA strand in the ternary complex structure of yeast RNAP-II. The conserved location of the Cα of R338 in the active site of PolD suggests that it is also involved in interacting with the DNA template. Second, PolD and two-DPBB polymerases share a loop that emanates from the C-terminal end of DPBB-1 (DPBB-B, in DNA-dependent RNAPs) and is connected to the adjacent DPBB through secondary structure interactions. In DNA-bound structures of both PolD and DNA-dependent RNAPs, this loop (hereafter named DPBB-connecting loop) hosts several well-conserved basic residues that contribute to the duplex-binding cleft. Indeed, DP2 R659, K661 residues occupy equivalent positions to the *S*. *cerevisiae* Rpb2 R1096, K1102, which bind the minor groove of the nascent RNA–DNA hybrid duplex. Third, both PolD and DNA-dependent RNAPs evolved a DNA-binding clamp domain, which occupies the same relative location with respect to the two-DPBB active site. In both enzymes, clamp domains host several loops that participate in binding the nascent DNA–DNA or RNA–DNA duplex and several zinc-binding domains (Zn-I and Zn-II domains in PolD; Zn-7, Zn-8, and Zn-6 in *S*. *cerevisiae* RNAP-II), which stabilize the loop-rich clamp domains. Whereas these clamps do not share sequence motifs, Zn-I of PolD and Zn-7 of RNAP-II share the same topology, which is conserved in all eukaryotic/archaeal DNA-dependent RNAPs. These structural elements are clustered in a region of their primary sequence that encompasses the two DPBBs (Fig 9C). Although these structural elements are carried on a single chain in PolD and QDE-1, these structural elements are hosted by two different subunits in multisubunit RNAPs, clustered at the C-terminal end of Rpb2 and the N-terminal end of Rpb1 in *S*. *cerevisiae* RNAP-II. This observation suggests that although all these two-DPBB polymerases share a conserved ancestor, diversification and complexification may have resulted in a scission of the minimal catalytic core of multisubunit RNAPs into two separate genes.

Comparing these structures also documents how this minimal catalytic core has recruited different domains in order to adapt to their biological functions. As an example, DNA-dependent RNAPs involved in DNA transcription have evolved the lid domain (absent in PolD and QDE-1), which is inserted in DPBB-B, to prevent the formation of an extended upstream RNA–DNA hybrid. In PolD, two ancestral ssDNA binding domains, OB and KH-like, have been recruited for DNA replication to guide the DNA template into the active sites. Comparing PolD with two-DPBBs also reveals structural determinants that are specific to DNAPs versus RNAPs. The catalytic site of all two-barrel RNAPs is characterized by two catalytic Mg^2+^ that are coordinated by 4 invariant aspartic residues (479DFDGD483 in RPB1 and D837 in RPB2 in *S*. *cerevisiae* RNAP-II) [53] (S4 Fig). Only two aspartic residues are strictly conserved in PolD: 956DGD958, respectively corresponding to 481DGD483 in *S*. *cerevisiae* RPB1, possibly explaining why no density accounting for the presence of Mg^2+^ has been observed so far in the active site of PolD, neither in the DNA-bound PolD cryo-EM structure nor in the DP2 individual crystal structure. Phosphates of the incoming nucleotide may thus be required in order to bind the two catalytic magnesium ions in the active site, as observed for other DNAPs [46]. Although conserved in all two-DPBB RNAPs [18, 54], the bridge helix is notably absent in PolD. Instead, the DNA template seems to be guided in the active site of PolD by a KH-like domain (Fig 9A and 9B). Both the bridge helix in RNAPs and the KH-like domain of PolD display a canonic motif of residues that are colocalized with respect to the DNA substrate, suggesting that they may play a similar role in guiding the DNA substrate into the active site (Fig 9B).

## Discussion

Although behaving in all aspects like a true polymerase involved in DNA replication, PolD differs structurally from all other known DNAPs [9–11]. First, the PolD two-DPBB catalytic core differs from both Klenow-like and Polβ-like folds found in all other families of DNAPs. Second, the Mre11-like DP1 nuclease differs from the DnaQ-like exonuclease domains found in most DNAPs showing proofreading activity [55]. Indeed, PDE domains are encountered in a wide range of hydrolases, but their dedication to DNAP-associated proofreading is specific to PolD. Third, the clamp and KH-like DNA-binding domains of PolD are structurally distinct from the palm, thumb, and finger domains of other DNAPs. The DNA-bound native PolD cryo-EM structure reveals for the first time how domains—up to now never observed as being involved in DNA replication, to our knowledge—collaborate together to form a DNA-binding site. The PolD native cryo-EM structure provides a structural rationale to explain why the association between DP1 and DP2 is required to rearrange the DP2 catalytic core in its active conformation to clamp the DNA duplex and to orient efficiently the DP1 proofreading active site with respect to the DNA substrate.

Comparing the structures of their DNA-binding sites enabled us to delineate a minimal catalytic core that is shared by all two-DPBB polymerases. This minimal core is remarkably versatile and is found to be associated to a wide range of structurally distinct DNA-binding domains, which contribute to the wide range of substrate specificities (DNA dependent or RNA dependent), activities (RNA and DNAPs), and functions (DNA transcription, replication, gene silencing) exhibited by two-DPBB polymerases. Two-DPBB cores share a common ancestor, which may have been endowed with nucleotide polymerization in early forms of life. The ability of this catalytic core to acquire novel nucleotide polymerization activities, like DNA replication and DNA transcription, may have facilitated RNA-world/DNA-world transition. We hypothesize that a two-DPBB ancestor was present and functional as an RNA replicase in an RNA world. The versatility of the two-DPBB fold may have allowed DNA replication and transcription to jointly evolve from a common catalytic core rather than being invented separately.

**Fig 10:**
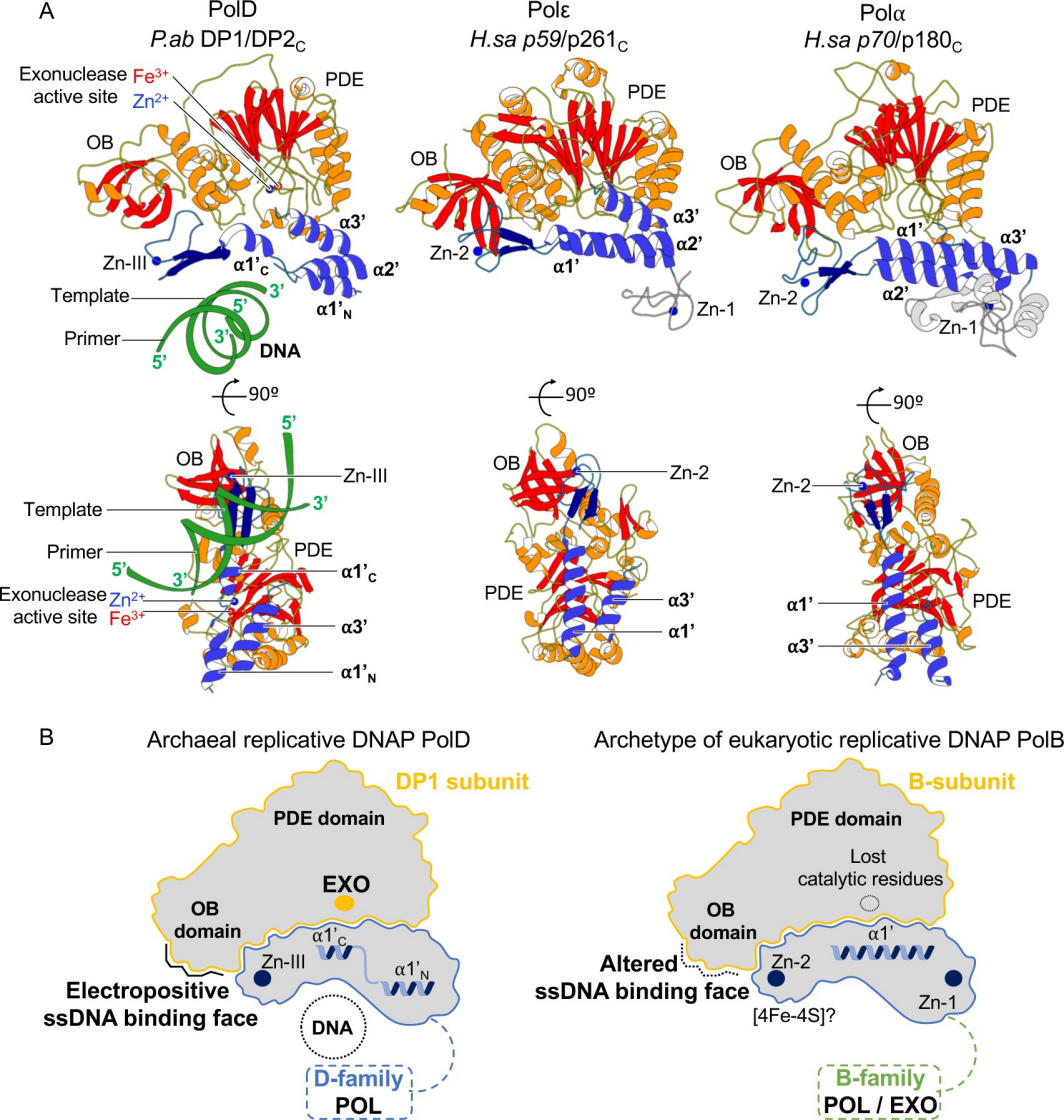
Structural comparison of the intersubunit interface of archaeal PolD and eukaryotic replicative DNAPs. (A) Comparison of the *P*. *abyssi* DP1/DP2–CTD region of the PolD cryo-EM structure, human Polε p59/p261_C_ (PDB ID: 5VBN [24]), and human Polα p70/p180_C_ (PDB ID: 4Y97 [58]) crystal structures. The eukaryotic Zn-1 binding domain that is not conserved in archaeal PolD is colored in gray. (B) Schematic representation of the DP1–DP2 interface of PolD and the A-subunit/B-subunit interface of eukaryotic DNAPs highlighting the differences between both structures. cryo-EM, cryo–electron microscopy; CTD, C-terminal domain; DNAP, DNA polymerase; EXO, exonuclease active site; *H*.*sa*, *H*. *sapiens*; OB, oligonucleotide binding; PDB, Protein Data Bank; PDE, phosphodiesterase domain; ssDNA, single-stranded DNA.

**Fig 11:**
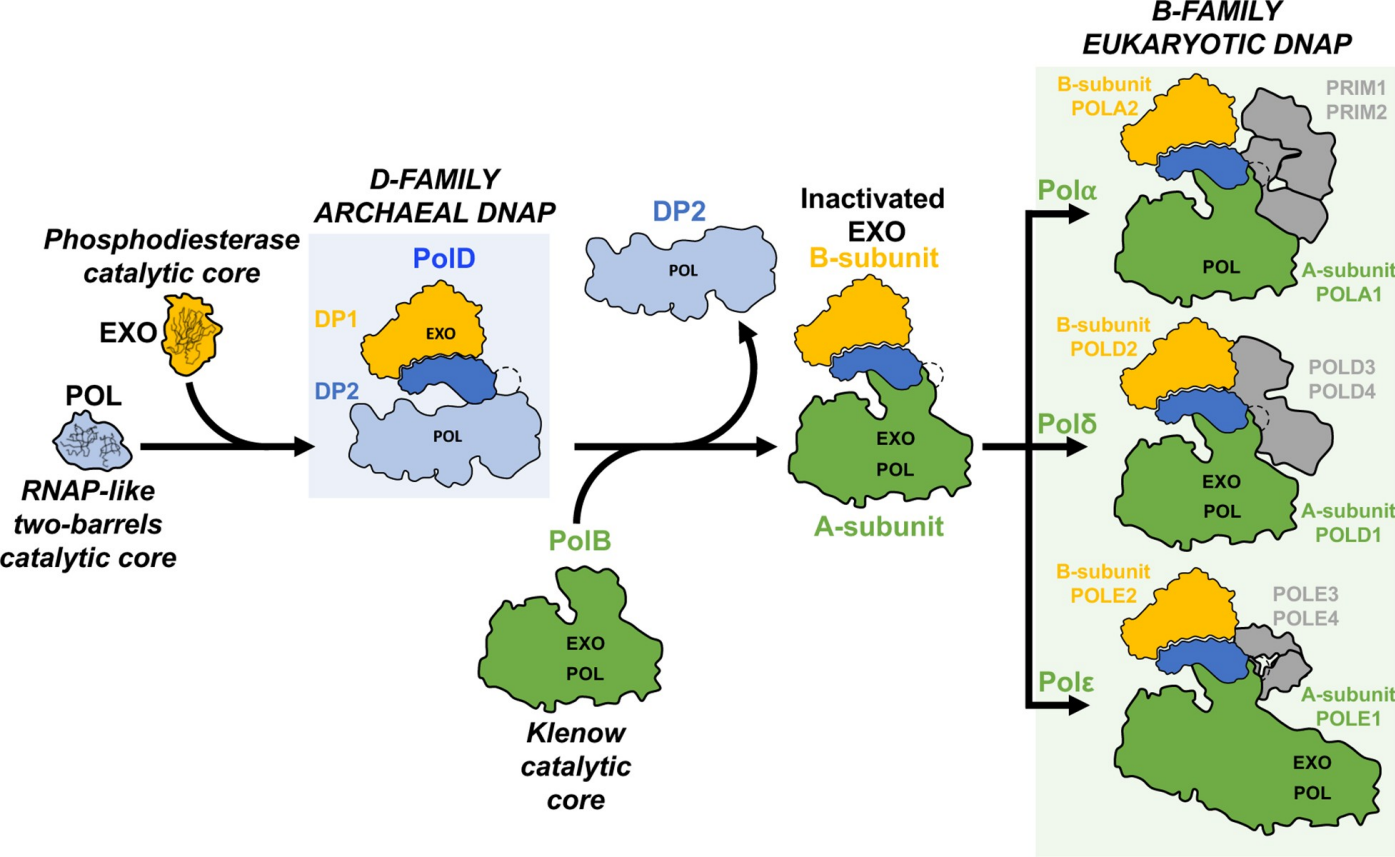
Hypothetical scenario accounting for the origins of archaeal PolD and its evolutionary relationships with the eukaryotic replicative DNAPs. Eukaryotic replicative DNAPs show a complex relationship with their archaeal ancestors, including contributions from both the B- and D-family of archaeal polymerases. During evolution, the D-family DP2 catalytic core was replaced by a B-family catalytic core in all eukaryotic replicative DNAPs, which are thus chimeric with respect to their archaeal homologues. Comparison of the PolD cryo-EM structure with the eukaryotic DNAPs shows that the eukaryotic A-subunit CTD/B-subunit complex has lost several features that are critical for the function of DP2–CTD/DP1 complex in PolD, suggesting the following hypothetical scenario that accounts for the evolutionary relationships between the archaeal PolD and the eukaryotic replicative DNAPs PolD has recruited an ancestral RNAP-like two-DPBB catalytic core for DNA replication. During evolution, the RNAP-like catalytic core of DP2 was substituted by a B-family-related catalytic subunit. The nuclease active site of DP1 was inactivated, as the proofreading activity is now carried on the catalytic subunit (A). While in PolD, the DP1/DP2–CTD complex contributes actively to DNA binding, and the eukaryotic A-subunit CTD/B-subunit complexes in Polα, Polδ, and Polε are involved in interacting with other members of the replication fork. cryo-EM, cryo–electron microscopy; CTD, C-terminal domain; DNAP, DNA polymerase; EXO, exonuclease active site; RNAP, RNA polymerase.

Eukaryotic replicative DNAPs show a complex relationship with their archaeal ancestors, including contributions from both the B- and D-families of archaeal polymerases. In addition to their catalytic subunit (A), all eukaryotic replicative DNAPs possess a regulatory B subunit. Bioinformatic predictions [56] and structural [15] studies have shown that DP1 is the ancestor of the B-subunits of eukaryotic multimeric DNAPs, which, however, lost the nuclease catalytic residues [56]. During evolution, the D-family DP2 catalytic core was replaced by a B-family catalytic core in all eukaryotic replicative DNAPs, which are thus chimeric with respect to their archaeal homologues (Fig 10). In addition, PolD and eukaryotic DNAPs share a conserved domain located in the C-terminal region of their catalytic subunits, which is dedicated to interacting with their DP1 or B subunit, respectively. The cryo-EM structure of PolD and crystal structures of the CTD/B-subunit complexes of Polα and Polε show a well-conserved Zn-binding domain (Zn-III in PolD, Zn-2 in eukaryotic DNAPs) that snugly fits a docking site located at the OB/PDE interface and a helical bundle that interacts with the PDE domain. Comparison with the eukaryotic DNAPs shows substantial structural differences that may account for functional ones. Indeed, the eukaryotic A-subunit CTD/B-subunit complex has lost several features that are critical for the function of the DP2–CTD/DP1 complex in PolD. All residues of the hydrolase active site of DP1 and all conserved basic residues hosted by the DP1 OB and DP2 clamp-1 domains are lost in eukaryotic DNAPs. In Polδ and Polε, proofreading activity is provided instead by their large A-catalytic subunit, which contains an exonuclease domain. Altogether, these observations suggest the following hypothetical scenario that accounts for the evolutionary relationships between the archaeal PolD and the eukaryotic replicative DNAPs (Fig 11). PolD has recruited an ancestral RNAP-like two-DPBB catalytic core for DNA replication. The capacity of PolD to use RNA-primed DNA and incorporate ribonucleotides may be a property inherited from its common ancestor to RNAP [57]. During evolution, the RNAP-like catalytic core of DP2 was substituted by a B-family related catalytic subunit. The nuclease active site of DP1 was inactivated, as the proofreading activity is now carried on the catalytic subunit (A). While in PolD, the DP1–DP2–CTD complex contributes actively to DNA binding, and the eukaryotic A-subunit CTD/B-subunit complexes in Polα, Polδ, and Polε are involved in interacting with other members of the replication fork.

## Methods

### Cloning, expression, and purification

The full sequences of *P*. *abyssi* PolD encoding the DP1 H451A and DP2 subunits were cloned into the RSF1-Duet expression vector (Novagen) fused to a 14-histidine tag with a TEV protease cleavable site at the DP1 N terminus. The full-length PolD entire complex was coexpressed by 1 mM IPTG induction in *E*. *coli* strain BL21 (DE3) Rosetta2 grown overnight in Lysogeny broth (LB) at 20°C and copurified by Ni-NTA and heparin chromatography (GE Healthcare), followed by TEV cleavage of the tag and size-exclusion chromatography. The purified PolD was concentrated to 2 mg/ml in 20 mM Tris HCl (pH 8), 200 mM NaCl, 3 mM MgCl_2_ storage buffer. PolD was flash-frozen in liquid nitrogen and stored at −80°C. DP2 (1–1061), the DP1 (144–619)–DP2 (1096–1195) complex, and DP1 H451A (144–619) were expressed and purified as previously described [15].

### Analytical ultracentrifugation assays

Sedimentation velocity experiment was performed with a Beckman Coulter XL-I analytical ultracentrifuge (Beckman Coulter, Indianapolis, IN, USA) with an An-60 Ti rotor at 20°C. The PolD complex at a concentration of 0.2, 0.5, and 1 mg/ml was centrifuged at 42,000 rpm in 12-mm double-sector epoxy centerpieces. Detection of the protein was performed both by absorbance at 280 nm and interferometry, and the ProteomeLab software (Beckman Coulter, Indianapolis, IN, USA) was used to monitor the experiment. Four hundred scans were collected at 3-min intervals with a radial step size of 0.003 cm. Profiles were analyzed using the continuous (s) distribution model of the software Sedfit [59]. The partial specific volume of the protein and the viscosity and density of the buffer at 20°C were theoretically calculated with the software Sednterp (Spin Analytical, Berwick, ME, USA).

### Biochemical primer-elongation assays

#### Oligonucleotides

Nucleotide sequences of the DNA substrates were inspired by Jokela and colleagues [60]: a 24-mer DNA primer (5′-ACGCCAGGCTTCGCCAGTCACGAC-3′) and a 60-mer DNA template (5′-GCGGACTGCGATCGTACCTACGGACCTGCAGCTGACGTCGTGACTGGCGAAGCCTGGCGT-3′). DNA primers were 5′-labeled with ^32^P, and the DNA duplex was annealed as described previously [15].

#### Activity tests

Five-micrometer native full-length DP1–DP2 PolD and 50-μM DP2(1–1061), 50 nM DNA duplex, 25 mM Tris HCl (pH 7.6), 25 mM NaCl, and 2 mM MgCl_2_ in presence of 200 nM dNTPs were incubated at 55°C for 1, 5, and 10 min. All reactions were stopped by adding formamide. Samples were run through a 15% acrylamide–8 M urea sequencing gel and revealed using a PhosphorImager Storm 860 (Fujifilm).

### DNA-binding assays by steady-state fluorescence anisotropy

#### Oligonucleotides

DNA substrates were obtained by mixing equimolar amounts of complementary strands (hexachlorofluorescein [HEX]-labeled DNA template: 3′-GCGGCCCGGCTCGGCACGTGCTGGA-5′-HEX, mixed with the DNA primer: 5′-CGCCGGGCCGAGCCGTGC-3′, or the full complementary: 5′-CGCCGGGCCGAGCCGTGCACGACCT-3′) in 20 mM Tris HCl (pH 8), 300 mM NaCl, and 1 mM EDTA and by heating at 95°C for 5 min, followed by slow cooling to room temperature.

#### DNA-binding assays

The binding of 3′-HEX-labeled DNA substrates by PolD and DP2 (1–1061) was determined by measuring the steady-state fluorescence anisotropy parameter using a spectrofluorometer equipped with polarizers (FL920, Edinburgh Instruments, Livingston, UK) in a cell thermostatically held at 25°C. The excitation and emission wavelengths were adjusted to 525 nm (2-nm bandpass) and 555 nm (20-nm bandpass), respectively. Titrations were performed in 20 mM sodium-succinate (pH 6), 100 mM NaCl by increasing concentrations of PolD up to 600 nM and DP2 (1–1061) up to 6 μM, to 5 nM of HEX-labeled DNA template. Details about steady-state fluorescence anisotropy calculation are mentioned in S1 Text.

### Sample preparation for cryo-EM

#### DNA duplex annealing

A unit of 100 μM of template: 5′-ACTTTGACGCGGCCCGTCTC-3′ was mixed with 100 μM of primer: 5′-GAGACGGGCCGCGTC-3′ in the annealing buffer: 20 mM Tris HCl (pH 8), 10 mM MgCl2, and 1 mM EDTA; incubated for 5 min at 95°C; and slowly cooled to room temperature. The duplex is formed by a 15-bp primed DNA substrate with a 5-nt overhang mimicking a replicated strand.

#### Grid preparation

The PolD protein complex (2 μM) was incubated for 10 min at 4°C with 3 μM of DNA before the samples were pipetted onto glow-discharged holey carbon cryo-EM grids (C-flat Cu 1.2/1.3, 300 mesh, Thick) and frozen in liquid ethane by using a Vitrobot Mark IV (ThermoFischer) at 100% humidity, 22°C temperature, and blotting time of 2 s.

### Cryo-EM data collection and image processing

Images of PolD in complex with DNA were collected using the EPU software on a Titan Krios electron microscope (ThermoFischer) operated at 300 kV and equipped with a K2 summit direct electron detector (Gatan, Pleasanton, CA) and a Bioquantum energy filter with 20-eV slit. Data were collected in single-electron counting mode at a nominal magnification of 105.000× (1.36 Å/pixel). The defocus range was set between −1 and −3 μm with a total dose of 60 electrons per Å^2^ over a total of 60 frames. The dose rate on the K2 camera was 4.729 e/pixel/s and the exposure time 24 s. Movie frame alignment with dose weighting and contrast transfer function (CTF) estimation was performed on the fly using a Scipion suite. Particle picking was performed using RELION-2.1.0 [22]; however, all subsequent classifications, generation of initial model, and 3D refinement are done using CryoSPARC-0.6.5. As illustrated in Fig 1C, about 200,000 particles were picked from 952 micrographs. Four rounds of referenced 2D alignments and classifications (25 iterations) were carried out. The 2D references were iteratively improved between the rounds. After four rounds of 2D classifications, spurious particles were removed to yield an overall dataset of 74,674 particles. The 74,674 particles from good 2D classes were selected for the unsupervised 3D classification using three classes, and three distinct groups of particles were identified: two groups representing the PolD complex without DNA and the third group (15.285) representing the PolD complex with DNA. The two first groups showed preferential orientation of the particles, whereas the third DNA-bound group showed a balanced angular distribution of the particles (S5 Fig). No structurally distinct groups of PolD emerged from subsequent 3D classifications, and the density corresponding to DNA is unequally well resolved from one subset of particles to the other, thereby suggesting partial occupancy and/or some flexibility of the DNA. The best density was obtained from a subset of 8,774 particles isolated from iterative 3D classifications, yielding to a density map at an overall resolution of 7.1 Å (Fig 1C and S1 Table). This 8,774-particle subset emerged from a previous 3D class (15,282 particles) that could be refined to 6.7 Å. Whereas this model displays a slightly higher resolution, the density corresponding to DNA is weaker, so it will not be discussed further. The overall resolution was estimated using the gold-standard FSC = 0.143 criterion. B-factor sharpening was performed using automatic procedures in CryoSPARC-v0.6.5 [23] (S1 Table). Local resolution for map was estimated by CryoSPARC-v0.6.5 [23].

### Building and refinement of the cryo-EM model

Individual crystal structures were manually placed in the cryo-EM maps and subsequently rigid-body fitted in the density using Coot [26, 61]. To ensure that the H451A mutation does not alter the structure of the DP1 subunit, the structure of the DP1 H451A (144–622) proofreading-deficient variant was solved using X-ray crystallography at 2.6-Å resolution. Neither the overall structure nor the coordination of the catalytic metal ions is altered in the DP1 H451 crystal structure (S2 Fig). The *P*. *abyssi* DP1 H451A (144–622) and *P*. *abyssi* DP2 (1–1061) (PDB ID: 5IJL [15]) crystal structures were divided into domains and subdomains to improve the precision of fitting. The DP1–DP2 rigid-body groups are listed in S6 Fig. Several densities not covered by the crystal structures were observed in the catalytic core of DP2 and at the interface between DP1 and DP2 subunits. The C-terminal region of DP2 (1090–1195) dedicated to interaction with DP1 was built by homology modeling with the CTD of the catalytic subunit of human Polε [24] (PDB ID: 5VBN) using Phyre [36]. The model was first to be rigid-body refined into the cryo-EM map and subsequently adjusted using modeling tools in Coot [26]. Several structural elements of the DP2 subunits that were not covered by the former DP2 (1–1061) crystal structure were built guided by the homology with the structure of yeast RNAP-II (PDB ID: 2E2I [25]). In addition, two α helices, α14 (324–338) and α43 (1074–1089), were built de novo in the cryo-EM density. The 15-mer/16-mer primer/template duplex B-form DNA was generated with Coot [26] and docked in the electron density, guided by the unambiguous density for the duplex region, showing minor and major grooves. No clear density is observed for the four 5′-terminal nucleotides of the template ssDNA, which were not included in the model. Details about the 3D model reconstructions are shown in Fig 2. All side chains of DP1 and most side chains of DP2 (86%) were deduced from the existing crystal structures; the others were modeled according to the most likely rotamers. The model was real-space refined using Phenix [62], with a high weight on ideal geometry and restraints on secondary structures (S1 Table). The final map correlation coefficient is 0.803.

### Structure determination of the DP1 H451A proofreading-deficient variant by X-ray crystallography

The structure of the DP1 H451A variant was determined by X-ray crystallography at 2.6 Å, following a similar procedure as that used for the wild-type DP1 crystal structure [15]. Details about crystallization, structure determination, model building, and refinement of the DP1 H451A variant are mentioned in S2 Text and S2 Table.

## Supporting information

S1 Table Values of cryo-EM data collection and 3D reconstruction. Cryo-EM, cryo–electron microscopy.
(DOCX)

S2 Table Data collection and refinement statistics. ^a^Numbers in parentheses refer to the highest-resolution shell. ^b^CC1/2 = percentage of correlation between intensities from random half-datasets. ^c^Calculated with MolProbity.
(DOCX)

S1 Text Details on anisotropy and Kd calculations for the DNA-binding assays by steady-state fluorescence anisotropy. (DOCX)

S2 Text Details on the structure determination of the DP1 H451A proofreading-deficient variant by X-ray crystallography. (DOCX)

S1 Fig FSC curves and local resolutions. (A) Gold-standard FSC curve calculated from CryoSPARC-0.6.5 for the map of the DNA-bound PolD showing no significant overfitting. Resolution is reported at FSC = 0.143. (B) Local resolution map of the DNA-bound PolD calculated from CryoSPARC-0.6.5. Surfaces are colored according to the resolution scale (right). The most resolved regions of the maps (local resolution around 5 Å) are the exonuclease and polymerase catalytic cores of the DP1 and DP2 subunits. FSC, Fourier shell correlation.
(DOCX)

S2 Fig Crystal structure of the proofreading-deficient H451A DP1 variant. (A) Comparison of the nuclease active sites of DP1 wild type (PDB ID: 5IHE) (left) and the proofreading DP1 H451A variant (PDB ID: 6HMF, this study) (right). Whereas H451 is a critical catalytic residue acting as a proton donor, the H451A mutation does not alter the binding of the divalent catalytic metals. The blue mesh shows the 2Fo-Fc electron density map contoured at 8.0 σ. (B) Superimposition of the DP1 wild-type (black) and DP1 H451A (yellow) overall structures. Both structures can be superimposed over 440 Cα with an r.m.s.d. of 0.079 Å. PDB, Protein Data Bank; r.m.s.d., root-mean-square deviation.
(DOCX)

S3 Fig Purification of the DP1_ΔN_(144–619)–DP2_CTD_(1096–1195) complex. (A) Gel-filtration chromatogram for the DP1_ΔN_ (144–619)/DP2_CTD_ (1096–1195) complex. (B) Fractions from the major peak of the gel filtration shown in (A) were subjected to SDS-PAGE and Coomassie blue staining. CTD, C-terminal domain; M, molecular weight markers.
(DOCX)

S4 Fig Structure-based alignment of the shared structural elements within the catalytic cores of PolD, DNA-dependent RNA polymerases, and RNA-dependent RNA polymerases. The sequences are denoted by the polymerase name and the abbreviated species names. The shared secondary structure elements are shown above the alignment; H indicates α helix, B indicates β strand, and L indicates loop. Yellow boxes highlight hydrophobic residues, green boxes highlight hydrophilic residues, and red boxes highlight catalytic motifs and metal ion–binding motifs. Regions of PolD that were built using homology modeling against the structures of DNA-dependent RNA polymerases are highlighted by a blue box. A, *Archaeoglobus*; E, *Escherichia*; H, *Haloferax*; K, *Korarchaeum*; Loki, Cand. Lokiarchaeon; M, *Methanomassiliicoccus*; N, *Neurospora*; P, *Pyrococcus*; S, *Saccharomyces*; S. shibatae, *Sulfolobus shibatae*; T, *Thermococcus*.
(DOCX)

S5 Fig Image processing of the cryo-EM 3D reconstruction of PolD. Workflow showing the evolution of the direction distributions along the iterative 3D classifications. Particles composing each class have been 2D classified. The 2D classification (selected 2D class averages are indicated), ab initio initial model reconstruction, 3D classification, refinement, and map sharpening are performed in CryoSPARC-0.6.5. DP1 and DP2 volumes are respectively represented in yellow and blue. The DNA duplex volume is represented in coral. cryo-EM, cryo–electron microscopy.
(DOCX)

S6 Fig List of rigid-body groups used for real-space refinement. (A) Crystal structures were divided into rigid-body groups in order to improve the precision of the fitting. Rigid-body groups were used for fitting and refining the DP1 and DP2 crystal structures in the cryo-EM map. (B) Different views of PolD showing the different rigid-body groups used during refinement. cryo-EM, cryo–electron microscopy.
(DOCX)

S1 Data (XLSX)

## Abbreviations

cryo-EM: cryo–electron microscopy
CTD: C-terminal domain
dGTP: deoxyguanosine triphosphate
DNAP: DNA polymerase
DPANN: Diapherotrites, Parvarchaeota, Aenigmarchaeota, Nanoarchaeota, Nanohaloarchaeota
DPBB: double-psi β-barrel
dsDNA: double-stranded DNA
GTPase: guanosine triphosphatase
HSH: helix-strand-helix
KH: K-homology
OB: oligonucleotide binding
PCNA: proliferating cell nuclear antigen
PDB: Protein Data Bank
PDE: phosphodiesterase domain
PIP: PCNA-interacting peptide
QDE-1: quelling defective phenotype
RFC: replication factor C
r.m.s.d.: root-mean-square deviation
RNAP: RNA polymerase
ssDNA: single-stranded DNA
TACK: Thaumarchaeota, Aigarchaeota, Crenarchaeota, Korarchaeota
