# Supplementary material for: Structure of the DP1–DP2 PolD complex bound with DNA and its implications for the evolutionary history of DNA and RNA polymerases
Source: PLoS Biol. 2019 Jan 18;17(1):e3000122. doi: 10.1371/journal.pbio.3000122 (PMC6355029; doi:10.1371/journal.pbio.3000122)
Supplement: S2 Table — aNumbers in parentheses refer to the highest-resolution shell. bCC1/2 = percentage of correlation between intensities from random half-datasets. cCalculated with MolProbity. (DOCX) [file pbio.3000122.s002.docx]

|  | **DP1 H451A** |
| --- | --- |
| **Data collection** |  |
| Space group | P2_1_2_1_2_1_ |
| Wavelength (Å) | 1.04 |
| Cell dimensions |  |
| a, b, c (Å) | 85.7, 91.2, 143.9 |
| α, β, γ (°) | 90, 90, 90 |
| Resolution (Å) | 48.0-2.60 (2.72-2.60)^a^ |
| Rsym (%)  I/σ(I) | 3.8 (64.7)  12.0 (1.1) |
| Completeness (%) | 100 (100) |
| Redundancy  CC_(1/2)_^b^ (%) | 8.5 (8.8)  99.6 (61.7) |
|  |  |
| **Refinement** |  |
| Resolution (Å) | 47.2-2.60 |
| No. reflections | 35447 |
| *R_work_/R_free_* | 22.9/25.5 |
| No. atoms |  |
| Protein | 7036 |
| Ions | 23 |
| Water | 63 |
| B-factors (Å^2^) |  |
| Protein | 81.2 |
| Ligand | 100.3 |
| Water | 74.4 |
| R.m.s deviations |  |
| Bond lengths (Å) | 0.009 |
| Bond angles (°) | 1.10 |
| Ramachandran |  |
| Favored/outliers (%)^c^ | 96.0/0.35 |
| Molprobity^4^ score | 1.60 |
|  |  |
|  |  |
