# Supplementary material for: Structure of the DP1–DP2 PolD complex bound with DNA and its implications for the evolutionary history of DNA and RNA polymerases
Source: PLoS Biol. 2019 Jan 18;17(1):e3000122. doi: 10.1371/journal.pbio.3000122 (PMC6355029; doi:10.1371/journal.pbio.3000122)
Supplement: S1 Text — (DOCX) [file pbio.3000122.s003.docx]

**S1 Text. Details on anisotropy and Kd calculations for the DNA-binding assays by steady-state fluorescence anisotropy.** Using a vertical direction for the polarized excitation source, the steady-state fluorescence anisotropy (*r*) was calculated according to the equation:

$$r=\frac{I_{V}-I_{H}}{I_{V}+2\times I_{H}}$$

where I_V_ and I_H_ correspond to the parallel (vertical) and perpendicular (horizontal) fluorescence emission intensity components, respectively.

The fraction of DNA/PolD complexes (i.e. the fraction of DNA bound to PolD), fb, was calculated using the following relationship:

$$fb=\frac{r-rf}{r-rf+(rb-r)\times(\frac{Ib}{If})}$$

where r is the measured fluorescence anisotropy value, rf and rb correspond to the fluorescence anisotropy values of free DNA (ligand in the free state, measured in the absence of PolD) and PolD-bound DNA (ligand in the bound state, corresponding to the maximum anisotropy value found at the plateau), respectively. This relationship takes into account the change in the fluorescence intensity of 3’-hexachorofluorescein (HEX)-labeled DNA observed along the titration (typically, the fluorescence intensity of HEX-labeled DNA in the bound state, Ib, was found to be 50% lower than the one characterizing the ligand in the free state, If). The Kd characterizing the DNA/PolD complex (Kd) was calculated by fitting the plot of fb versus [PolD] with a Hill model using the IgorPro Software (Wavemetrics).
