# Supplementary material for: Structure of the DP1–DP2 PolD complex bound with DNA and its implications for the evolutionary history of DNA and RNA polymerases
Source: PLoS Biol. 2019 Jan 18;17(1):e3000122. doi: 10.1371/journal.pbio.3000122 (PMC6355029; doi:10.1371/journal.pbio.3000122)
Supplement: S2 Text — (DOCX) [file pbio.3000122.s004.docx]

**S2 Text. Details on the structure determination of the DP1 H451A proof-reading deficient variant by X-ray crystallography.** DP1 H451A was crystallized by hanging-drops vapour diffusion at 4°C, mixing 1 μl of protein (3 mg/ml) with 1 μl of crystallization buffer containing 100 mM sodium cacodylate, pH 6.7, 200 mM calcium acetate and 2-6% PEG8000. Crystals were cryo-cooled with 27.5% ethylene glycol. Crystals belonged to the P2_1_2_1_2_1_ space group with two copies of the protein in the asymmetric unit and diffracted up to 2.6 Å resolution on ID29-1 (ESRF, Grenoble, France). The structure was determined by molecular replacement using the crystal structure of DP1 wild-type^1^. The model was modified manually in Coot^2^ and refined in Buster^3^. The final model was refined in Buster to R/Rfree values of 22.9/25.5% at 2.6 Å resolution. Non-crystallographic symmetry restraints were used throughout refinement, with one TLS parameter per molecule. Residues 144-151, 164-173, 213-224 and 514-517 (only in chain B), which were not visible in the electron density, were not included in the final model. One molecule of cacodylate per monomer and and 67 water molecules were added. In the final electron density, remaining peaks that could not be attributed to water were modelled with acetate, and one calcium ion, which were present in the crystallization solution (see above). The active site includes two tightly-bound metal ions, zinc and iron, whose presence was previously confirmed by collecting anomalous data at the zinc and iron K-edges^1^. 96 % of residues were in the favoured regions of the Ramachandran plot with three outliers. Two out of these three outliers correspond to His residues that participate to metal ion coordination and are located in the active site in a region where the electron density is clearly defined. Metal ion coordination causes a subtle distortion of the backbone resulting with these residues being Ramachandran outliers. The Molprobity^4^ score for the refined model is 1.6, in the 99^th^ percentile of structures refined at comparable resolution. Dali^5^ was used to compare the PolD structure with those of the Protein Data Bank. Electrostatic surface potentials were calculated using APBS^6^ in Chimera^7^. The evolutionary conservation analysis of surface residues was performed with the Consurf^8^ server. Figures were prepared with Chimera^7^ and PyMOL (The PyMOL Molecular Graphics System, Version 1.8 Schrödinger, LLC.).

**References:**

1. Sauguet, L., Raia, P., Henneke, G. & Delarue, M. Shared active site architecture between archaeal PolD and multi-subunit RNA polymerases revealed by X-ray crystallography. *Nat. Commun.* **7**, 12227 (2016).

2. Emsley, P. & Cowtan, K. Coot: model-building tools for molecular graphics. *Acta Crystallogr. D Biol. Crystallogr.* **60**, 2126–2132 (2004).

3. Blanc, E. *et al.* Refinement of severely incomplete structures with maximum likelihood in BUSTER-TNT. *Acta Crystallogr. D Biol. Crystallogr.* **60**, 2210–2221 (2004).

4. Davis, I. W. *et al.* MolProbity: all-atom contacts and structure validation for proteins and nucleic acids. *Nucleic Acids Res.* **35**, W375–383 (2007).

5. Holm, L. & Rosenström, P. Dali server: conservation mapping in 3D. *Nucleic Acids Res.* **38**, W545–W549 (2010).

6. Baker, N. A., Sept, D., Joseph, S., Holst, M. J. & McCammon, J. A. Electrostatics of nanosystems: application to microtubules and the ribosome. *Proc. Natl. Acad. Sci. U. S. A.* **98**, 10037–10041 (2001).

7. Pettersen, E. F. *et al.* UCSF Chimera--a visualization system for exploratory research and analysis. *J. Comput. Chem.* **25**, 1605–1612 (2004).

8. Landau, M. *et al.* ConSurf 2005: the projection of evolutionary conservation scores of residues on protein structures. *Nucleic Acids Res.* **33**, W299–302 (2005).
