# Supplementary material for: Structure of the DP1–DP2 PolD complex bound with DNA and its implications for the evolutionary history of DNA and RNA polymerases
Source: PLoS Biol. 2019 Jan 18;17(1):e3000122. doi: 10.1371/journal.pbio.3000122 (PMC6355029; doi:10.1371/journal.pbio.3000122)
Supplement: S2 Fig — (A) Comparison of the nuclease active sites of DP1 wild type (PDB ID: 5IHE) (left) and the proofreading DP1 H451A variant (PDB ID: 6HMF, this study) (right). Whereas H451 is a critical catalytic residue acting as a proton donor, the H451A mutation does not alter the binding of the divalent catalytic metals. The blue mesh shows the 2Fo-Fc electron density map contoured at 8.0 σ. (B) Superimposition of the DP1 wild-type (black) and DP1 H451A (yellow) overall structures. Both structures can be superimposed over 440 Cα with an r.m.s.d. of 0.079 Å. PDB, Protein Data Bank; r.m.s.d., root-mean-square deviation. (DOCX) [file pbio.3000122.s006.docx]

**S2 Figure**
