# Supplementary material for: Structure of the DP1–DP2 PolD complex bound with DNA and its implications for the evolutionary history of DNA and RNA polymerases
Source: PLoS Biol. 2019 Jan 18;17(1):e3000122. doi: 10.1371/journal.pbio.3000122 (PMC6355029; doi:10.1371/journal.pbio.3000122)
Supplement: S3 Fig — (A) Gel-filtration chromatogram for the DP1ΔN (144–619)/DP2CTD (1096–1195) complex. (B) Fractions from the major peak of the gel filtration shown in (A) were subjected to SDS-PAGE and Coomassie blue staining. CTD, C-terminal domain; M, molecular weight markers. (DOCX) [file pbio.3000122.s007.docx]

**S3 Figure**
