# Supplementary material for: Structure of the DP1–DP2 PolD complex bound with DNA and its implications for the evolutionary history of DNA and RNA polymerases
Source: PLoS Biol. 2019 Jan 18;17(1):e3000122. doi: 10.1371/journal.pbio.3000122 (PMC6355029; doi:10.1371/journal.pbio.3000122)
Supplement: S4 Fig — The sequences are denoted by the polymerase name and the abbreviated species names. The shared secondary structure elements are shown above the alignment; H indicates α helix, B indicates β strand, and L indicates loop. Yellow boxes highlight hydrophobic residues, green boxes highlight hydrophilic residues, and red boxes highlight catalytic motifs and metal ion–binding motifs. Regions of PolD that were built using homology modeling against the structures of DNA-dependent RNA polymerases are highlighted by a blue box. A, Archaeoglobus; E, Escherichia; H, Haloferax; K, Korarchaeum; Loki, Cand. Lokiarchaeon; M, Methanomassiliicoccus; N, Neurospora; P, Pyrococcus; S, Saccharomyces; S. shibatae, Sulfolobus shibatae; T, Thermococcus. (DOCX) [file pbio.3000122.s008.docx]

**S4 Figure**
