# Supplementary material for: Structure of the DP1–DP2 PolD complex bound with DNA and its implications for the evolutionary history of DNA and RNA polymerases
Source: PLoS Biol. 2019 Jan 18;17(1):e3000122. doi: 10.1371/journal.pbio.3000122 (PMC6355029; doi:10.1371/journal.pbio.3000122)
Supplement: S5 Fig — Workflow showing the evolution of the direction distributions along the iterative 3D classifications. Particles composing each class have been 2D classified. The 2D classification (selected 2D class averages are indicated), ab initio initial model reconstruction, 3D classification, refinement, and map sharpening are performed in CryoSPARC-0.6.5. DP1 and DP2 volumes are respectively represented in yellow and blue. The DNA duplex volume is represented in coral. cryo-EM, cryo–electron microscopy. (DOCX) [file pbio.3000122.s009.docx]

**S5 Figure**
