# Supplementary material for: Structure of the DP1–DP2 PolD complex bound with DNA and its implications for the evolutionary history of DNA and RNA polymerases
Source: PLoS Biol. 2019 Jan 18;17(1):e3000122. doi: 10.1371/journal.pbio.3000122 (PMC6355029; doi:10.1371/journal.pbio.3000122)
Supplement: S6 Fig — (A) Crystal structures were divided into rigid-body groups in order to improve the precision of the fitting. Rigid-body groups were used for fitting and refining the DP1 and DP2 crystal structures in the cryo-EM map. (B) Different views of PolD showing the different rigid-body groups used during refinement. cryo-EM, cryo–electron microscopy. (DOCX) [file pbio.3000122.s010.docx]

**S6 Figure**
