# Supplementary material for: Structure of the DP1–DP2 PolD complex bound with DNA and its implications for the evolutionary history of DNA and RNA polymerases
Source: PLoS Biol. 2019 Jan 18;17(1):e3000122. doi: 10.1371/journal.pbio.3000122 (PMC6355029; doi:10.1371/journal.pbio.3000122)
Supplement: Supplementary file 9 [file pbio.3000122.s001.docx]

	DNA-bound PolD
Data collection	
Camera	Gatan K2
Voltage (kV)	300
Nominal magnification	105000x
Electron exposure (e-/Å2)	60
Defocus range (µm)	1.0-3.0
Pixel size (Å)	1.36
3D reconstruction	
Symmetry imposed	C1
Initial particle images (no.)	74674
Final particle images (no.)	8774
Map resolution (Å)<br>0.143 FSC threshold	7.1
Map sharpening B-factor (Å2)	362.8
